# Molecular mechanisms underlying cyclophosphamide-induced ovarian injury and protective strategies

**DOI:** 10.1007/s00210-025-04550-9

**Published:** 2025-09-12

**Authors:** Ehab E. Sharata, Taha Bakry, Habiba Gamal Atta, Habiba Atef Mohammed, Nazema Shaker Diab, Rofaida Ashraf Atef, Roaa Sayed Hosney, Mahmoud Mohamed Omar, Ramadan A. M. Hemeida

**Affiliations:** 1 Department of Pharmacology & Toxicology, Faculty of Pharmacy, Deraya University, Minia, 61111 Egypt; 2 Department of Pharmaceutics and Pharmaceutical Technology, Deraya University, 61519 Minia, Egypt

**Keywords:** Cyclophosphamide, Premature ovarian failure, Apoptosis, Inflammation, Oxidative stress

## Abstract

Cyclophosphamide (CP) is an anti-cancer medication that also treats chronic inflammatory illnesses caused by the immune system. Although CP is widely used, it can occasionally have limited therapeutic efficacy due to its significant combined toxicities. Ovarian damage caused by CP is a major problem for patients, and premature ovarian failure (POF) is a serious side effect of CP that commonly affects female patients. Mechanistic investigations have implicated oxidative stress, inflammatory responses, and apoptosis as critical components in the etiology of CP-induced POF, although the exact process by which this ovarian toxicity occurs remains unclear. After CP causes ovarian cells to generate proinflammatory cytokines, including interleukin-6 (IL-6) and tumor necrosis factor-alpha (TNF-α), nuclear factor kappa B (NF-κB) is activated. The activation of the NLRP3 inflammasome is the subsequent stage. In addition, Nrf2/HO-1 has been identified as an important signaling pathway that mitigates oxidative stress in CP-induced POF due to its anti-inflammatory and antioxidative characteristics. Moreover, several recent studies highlighted the role of α-klotho deficiency in ovarian aging. Quercetin, resveratrol, berberine, curcumin, irbesartan, mirtazapine, sildenafil, atorvastatin, donepezil, cilostazol, moxibustion, LCZ696, buspirone, levomilnacipran, melatonin, diosmin, and azilsartan are some of the agents that may protect against ovarian injury caused by CP, as shown in Graphical abstract. Our goal in writing this study is to provide a concise overview of the possible redox molecular pathways that cause ovarian harm in CP and how to potentially ameliorate them. Finally, investigation into these molecular pathways may pave the way for early ovarian damage relief and for the development of different agent strategies to alleviate CP-mediated POF.

**Graphical Abstract:**

**Figure:**
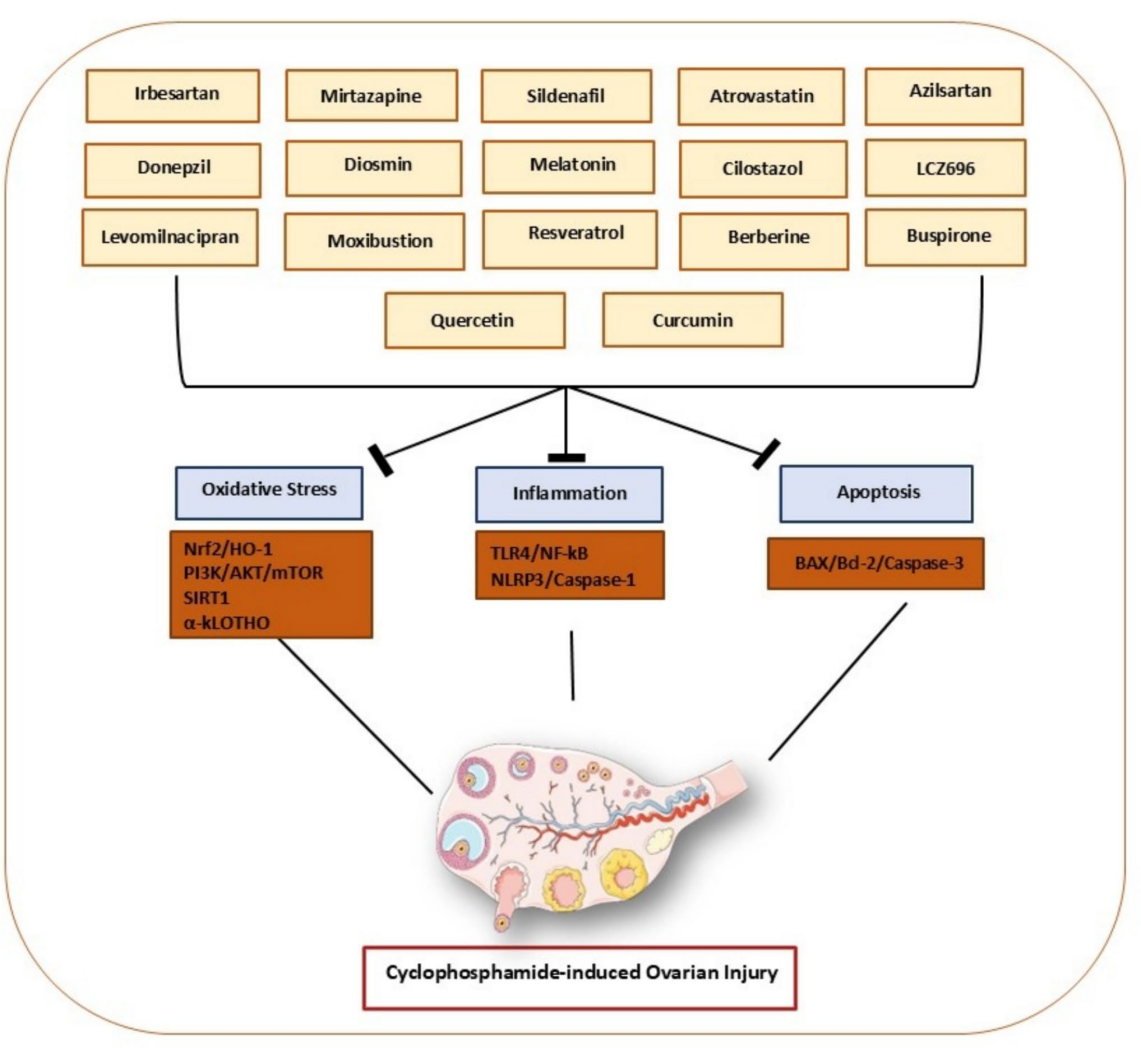
Schematic illustration summarizing the molecular mechanisms underlying cyclophosphamide-induced ovarian injury and the protective strategies of various pharmacological and natural agents. The illustration demonstrates how the specified agents provide their protective effects by targeting critical pathogenic pathways associated with cyclophosphamide-induced ovarian damage

## Introduction

Premature ovarian failure (POF), also known as primary ovarian insufficiency or early menopause, is associated with the cessation of ovarian Function before the age of 40 (Fu et al. 2021). The three criteria that characterize POF are amenorrhea persisting for a minimum of 4 months, diminished serum estradiol levels, and increased follicle-stimulating hormone (FSH) serum levels, which must exceed 40 IU/l in at least two samples taken several weeks apart (Mauri et al. 2020). Researchers estimate the incidence of POF to be 0.1% among women under 30 and 1% among women under 40 who develop spontaneous POF (Mauri et al. 2020). The aggregated prevalence of POF reached 3.7%, as indicated by a recent meta-analysis examining the global incidence of POF and early menopause (Rahman and Panay 2021). Radiation therapy and chemotherapy employed in cancer treatment are among the primary causes of POF. While chemotherapy and radiotherapy can improve cancer outcomes and promote prolonged lifespan in the young population (Zamanian et al. 2024; Golmohammadi et al. 2023), problems such as ovarian failure may occur (Zhao et al. 2023). The subsequent chemotherapy drugs are strongly correlated with ovarian failure in clinical contexts: cyclophosphamide (CP), methotrexate, and cisplatin (Bhardwaj et al. 2023; Chen et al. 2023; Li et al. 2023a). Ovarian function, characterized by rapid cellular turnover, may resemble tumor cells, as both are significant targets for chemotherapeutic agents. Ovarian primordial follicular cells lack regenerative capacity, and their loss leads to ovarian dysfunction, manifesting as POF and infertility due to oocyte depletion, along with DNA damage and alterations in the functional and structural properties of oocytes (Pouladvand et al. 2024).

### Pharmacological and clinical applications of cyclophosphamide

The FDA indicates that CP is primarily used for the treatment of malignant lymphomas in stages III and IV, as classified by the Ann Arbor staging criteria. These may encompass Hodgkin and non-Hodgkin lymphoma, lymphocytic lymphoma, small lymphocytic lymphoma, Burkitt lymphoma, and multiple myeloma (Mills et al. 2019; Xu et al. 2013; Ansell 2015). In Hodgkin lymphoma, CP has been utilized in specific combination regimens, particularly for patients who may be intolerant to traditional protocols (Ansell 2015). In non-Hodgkin lymphoma, CP is a core drug in several first-line regimens, especially B-cell lymphomas (Roschewski et al. 2014; Wang and Li 2021). High-dose CP is occasionally administered to individuals with refractory non-Hodgkin lymphoma and may facilitate transition to stem cell transplantation. Low-dose metronomic CP (continuous low dosing) has demonstrated potential for fragile individuals with low-grade non-Hodgkin lymphoma, providing effective results with reduced toxicity (Michot et al. 2020). CP has demonstrated a clinically and statistically significant advantage in diminishing disease activity in rheumatoid arthritis, enhancing tender and swollen joint scores, and lowering the incidence of new or exacerbated joint erosions (Suarez-Almazor et al. 1996). CP attenuates the immune system by suppressing lymphocyte (T and B cell) growth, hence diminishing inflammation and the autoimmune mechanisms associated with rheumatic illnesses (Klein et al. 2018). Additionally, CP is extensively utilized in the management of systemic lupus erythematosus with significant organ involvement (e.g., lupus nephritis), systemic vasculitis, including anti-neutrophil cytoplasmic antibody (ANCA)-associated vasculitis, systemic sclerosis with pulmonary involvement, and myopathies (Suarez-Almazor et al. 1996). CP in rheumatology may be administered orally or intravenously; intravenous pulse therapy (e.g., monthly infusions) is frequently employed to mitigate cumulative toxicity (Suarez-Almazor et al. 1996). It is typically utilized for a brief duration (3 to 6 months) to induce remission. Weaker drugs are then employed for maintenance after disease control is attained (Hudson et al. 2020). In nephrology, CP is a powerful immunosuppressive drug utilized for many severe kidney disorders, predominantly those of autoimmune origin. In rapidly progressive glomerulonephritis, CP is a primary therapeutic drug, frequently used alongside corticosteroids, to achieve remission in severe, life-threatening glomerulonephritis, including ANCA-associated vasculitis and Goodpasture’s syndrome (Ponticelli et al. 2018). In neurology, CP functions as a second- or third-line immunosuppressant for severe neuroimmunological disorders, especially when first-line therapies have proven ineffective. In CNS vasculitis, CP effectively induces remission in severe cases of primary or secondary vasculitis involving the brain (Osen et al. 2023; Nicole and Timothy 2024). In severe multiple sclerosis, CP is used for refractory or aggressive demyelinating disease. High-dose protocols can stabilize disease, reduce relapses, and sometimes improve function (Osen et al. 2023; Fereidan-Esfahani and Tobin 2021). It is considered in autoimmune encephalitis when standard immunotherapies are inadequate, particularly if there is a progressive course or relapse, and also used in neurosarcoidosis, some inflammatory neuropathies, and myasthenia gravis associated with immune checkpoint inhibitors (Nicole and Timothy 2024).

### Chemical properties and metabolism of cyclophosphamide

**Fig. 1:**
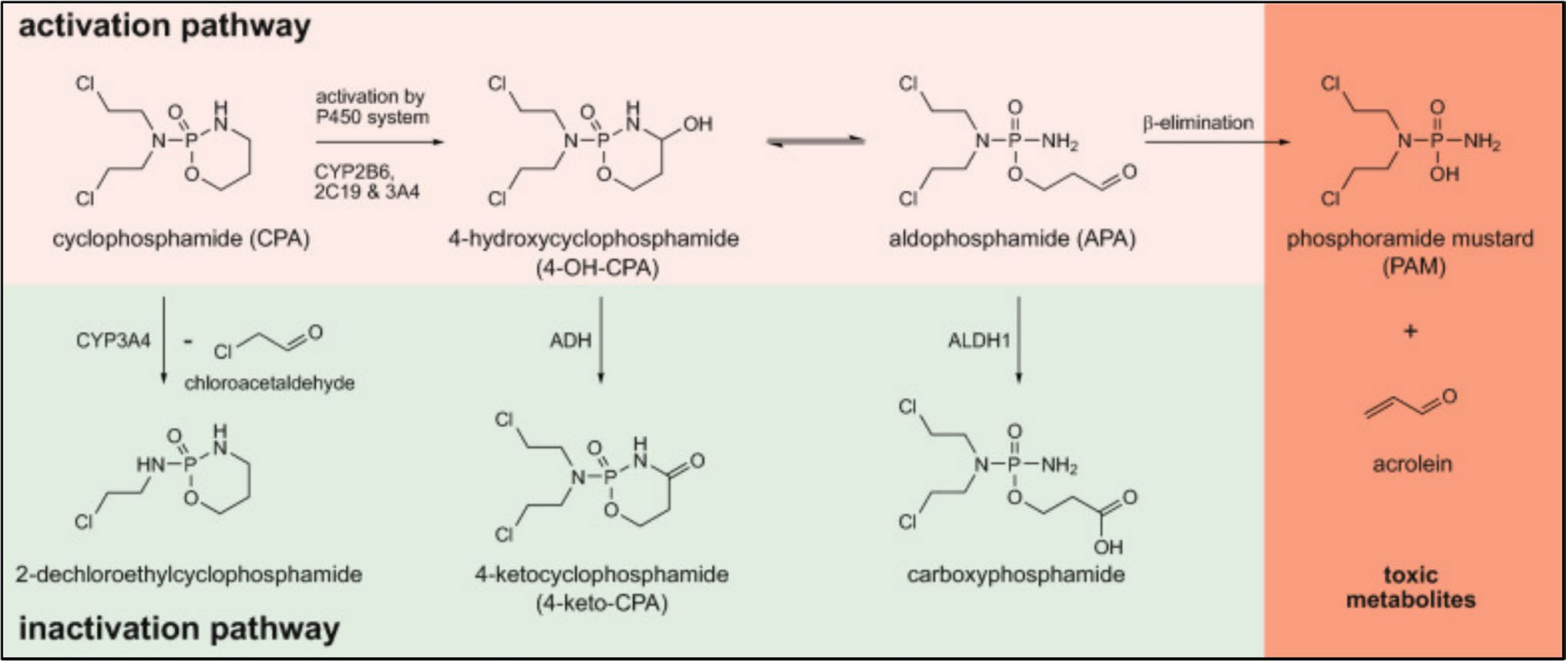
Metabolism of cyclophosphamide, including metabolic activation and inactivation pathways of cyclophosphamide (Steinbrecht et al. 2020)

CP ranks among the most efficacious anti-cancer drugs. CP remains employed as a chemotherapeutic agent in many malignancies and autoimmune disorders (Souza et al. 2024). The preliminary clinical trials of CP for cancer treatment commenced in 1958, and in 1959, it was sanctioned as the eighth cytotoxic anticancer agent by the FDA. CP functions as an inactive prodrug, necessitating enzymatic and chemical activation; the resulting nitrogen mustard induces interstrand and intrastrand DNA crosslinks, which are responsible for its cytotoxic effects (Barnes et al. 2024). CP, in combination with other antineoplastic agents, is utilized in the treatment of several cancers, including breast, lymphoid, and pediatric malignancies. CP is employed in bone marrow transplantation. Bone marrow suppression is the primary unfavorable effect of CP. Leukopenia, thrombocytopenia, and anemia commonly arise following the administration of high-dose CP (Scorer et al. 2023). Hemorrhagic cystitis is the primary sign of CP bladder toxicity; however, bladder fibrosis and transitional or squamous cell carcinoma may also develop. Hemorrhagic cystitis may present either acutely or chronically after chemotherapy treatment (Scorer et al. 2023). The pharmacological precursor CP is metabolized by cytochrome P450 enzyme systems in the Liver, generating 4-hydroxycyclophosphamide and aldophosphamide. Glycoproteins enable the active transport of these substances into the cell. Aldophosphamide is then transformed into the active metabolites phosphoramide and acrolein. Acrolein and phosphamide trigger DNA breaks by chemically binding to DNA (Steinbrecht et al. 2020). 4-Hydroxycyclophosphamide is enzymatically converted into 4-ketocyclophosphamide, while aldehyde dehydrogenase converts aldophosphamide into the innocuous carboxyphosphamide. Glutathione, an antioxidant, stabilizes carboxyphosphamide (Bignold 2006; Iqubal et al. 2019). Increased levels of 4-hydroxycyclophosphamide and carboxyethyl phosphamide have been detected in the blood plasma of individuals receiving CP therapy (Campagne et al. 2020). The primary metabolite, phosphoramide mustard, is a nitrogen mustard molecule considered the most therapeutically effective metabolite owing to its significant ability to induce DNA damage by intercalating between the two strands (Vredenburg et al. 2015). The second metabolite, acrolein, is produced when 4-hydroxy cyclophosphamide is activated to form active phosphoramide. It is a highly reactive metabolite considered the toxic element responsible for most of the deleterious effects of CP. It achieves this by impairing the body’s antioxidant defense systems and producing reactive oxygen species (ROS), including hydrogen peroxide and superoxide radicals. (Jeelani et al. 2017). ROS cause DNA and cellular membrane damage, inhibit specific enzymes, and facilitate lipid peroxidation, hence increasing the likelihood of infertility (Sun et al. 2015). Females subjected to CP and acrolein may encounter ovarian failure and infertility (Detti et al. 2013). The cytotoxicity of acrolein frequently restricts the clinical utilization of CP. Acrolein has been linked to oxidative stress generated by CP in animal models, which disrupts biochemistry and physiology by producing free radicals (Wahlang et al. 2015). The metabolism of CP is summarized in Fig. 1 (Steinbrecht et al. 2020).

The primary aim of this study is to elucidate the molecular mechanisms underlying CP-induced ovarian injury, with a focus on oxidative stress, inflammatory signaling, and apoptotic pathways, focusing on nuclear factor-kappa B (NF-κB), nuclear factor erythroid-2-related factor 2/heme oxygenase-1 (Nrf2/HO-1), Toll-like receptor 4 (TLR4), nucleotide-binding oligomerization domain-like receptor family pyrin domain-containing 3 (NLRP3) inflammasome, silent information regulator-1 (SIRT1), and other signaling pathways involved in the pathogenesis of ovarian injury caused by CP. Additionally, this review aims to critically evaluate various pharmacological and natural protective agents that have shown promise in experimental models to mitigate CP-mediated ovarian toxicity. Through integrating these molecular insights and protective strategies, we seek to highlight potential therapeutic targets and foster the development of effective interventions to preserve ovarian function in female patients undergoing chemotherapy.

### Fertility preservation strategies in clinical settings

#### Hormone replacement treatment

Hormone replacement treatment (HRT) is fundamental in the management of women with POF, including cases caused by chemotherapy and other pharmacological drugs. The principal objective of HRT is to substitute inadequate ovarian hormones, primarily estrogen, with or without progestin, to re-establish physiological levels closer to those observed before menopause. HRT does not serve as a means of fertility preservation for chemotherapy-induced POF. HRT serves as a method for symptom management and a long-term health strategy following ovarian injury (Cattoni et al. 2021; Webber et al. 2017).

HRT mitigates symptoms of estrogen shortage (such as hot flashes, vaginal dryness, sleeplessness, mood changes, and bone loss) and safeguards long-term health, mitigating the risks of osteoporosis and cardiovascular disease, while preserving uterine health when the endometrium is intact (Laml et al. 2000). HRT (estrogen + progesterone) enhances endometrial thickness, promoting an optimal condition for embryo implantation during in vitro fertilization or rare spontaneous pregnancies. By mimicking normal hormonal variations, HRT optimizes the endometrial cycle, facilitating scheduled embryo transfer (Cartwright et al. 2016; Dragojević-Dikić et al. 2009). In HRT, estrogen is delivered as transdermal estradiol (100 μg/day) or oral estradiol (2–4 mg/day) on days 1–26 of the cycle. Micronized progesterone (200 mg/day) or medroxyprogesterone acetate (10 mg/day) should be administered from days 16 to 26 to avoid endometrial hyperplasia (Fish 2011; Cartwright et al. 2016). Estrogen-progestin HRT elevates breast cancer risk by approximately 1.3 times after more than 5 years (Cartwright et al. 2016; Kou et al. 2016).

#### Gonadotropin-releasing hormone agonists

Gonadotropin-releasing hormone (GnRH) analogs, particularly agonists, are extensively researched for their ability to protect ovarian function during gonadotoxic therapies, such as chemotherapy, which may result in POF. Their main objective is to avert or diminish ovarian damage, hence saving future reproductive potential (Blumenfeld 2019a; Blumenfeld and Wolff 2008). GnRH agonists are not recognized as a main method for fertility preservation in spontaneous POF due to the lack of functioning follicles. Their role connects with POF settings. GnRH agonists, such as leuprolide, initially induce a “flare effect,” subsequently leading to pituitary desensitization and suppression of FSH and LH. This diminishes ovarian activity, apparently saving follicles from harm during chemotherapy. GnRH agonists may protect follicles from apoptosis following cytotoxic therapy by inducing a brief prepubertal hormonal state. Reducing ovarian perfusion, therefore, restricts chemotherapeutic exposure to the ovary, mitigating apoptosis in ovarian cells via the activation of intra-ovarian protective factors (Yuan et al. 2022). GnRH agonists are not used as treatment for confirmed POF. Their function is prophylactic, so they should be provided before and during cytotoxic therapy to mitigate the chance of developing POF. Clinical trials and meta-analyses indicated that concurrent administration of GnRH agonists during chemotherapy markedly reduced the incidence of POF and enhanced the likelihood of restarting menstruation and ovarian function following treatment (Blumenfeld and Wolff 2008; Blumenfeld et al. 2014; Blumenfeld 2019b).

#### Oocyte cryopreservation

Oocyte cryopreservation, sometimes referred to as egg freezing, is a medical process that involves the collection, fast freezing, and storage of a woman’s eggs (oocytes) for future utilization. This procedure is a crucial technique employed to maintain fertility (Han and Seifer 2023). Oocyte cryopreservation is a recognized method for maintaining fertility in women susceptible to POF. This method is particularly crucial for women undergoing gonadotoxic medications, possessing genetic predispositions, or experiencing medical disorders that may diminish ovarian reserve (Pai et al. 2021). Oocyte cryopreservation is useful for early-stage POF in women with irregular ovulation and a detectable antral follicle count or anti-Müllerian hormone (AMH), particularly those with hereditary predispositions (Oktay and Bedoschi 2014), autoimmune, or iatrogenic POF (González et al. 2012; Tomasi-Cont et al. 2014). The procedure of oocyte cryopreservation transpires through multiple stages. In initial ovarian stimulation, the female receives hormonal treatment to produce numerous mature oocytes. Subsequently, egg retrieval occurs, during which mature oocytes are extracted from the ovaries via a small surgical intervention. After cryopreservation, the harvested oocytes are often frozen using a method known as vitrification, which inhibits ice crystallization within the cells. Ultimately, the frozen eggs are preserved in liquid nitrogen until the mother decides to utilize them. In the future, when pregnancy is requested, the ova are thawed, fertilized with sperm in vitro, and subsequently implanted as embryos into the uterus (Han and Seifer 2023; Han and Seifer 2023).

## Molecular mechanisms promoting cyclophosphamide-induced ovarian injury

CP induces ovarian damage primarily through oxidative stress, inflammation, and apoptosis. Its bioactivation leads to excessive generation of ROS, including superoxide (O₂•⁻), hydrogen peroxide (H₂O₂), singlet oxygen, and hydroxyl radicals (•OH), disrupting redox homeostasis in ovarian tissue (Trujillo et al. 2023). CP also elevates nitric oxide (NO) levels, which rapidly react with superoxide to form peroxynitrite (ONOO⁻), a highly reactive oxidant that intensifies cellular injury (Khan et al. 2016; Shaeib et al. 2016). This oxidative burden impairs mitochondrial function and depletes antioxidant defenses, creating a self-propagating cycle of damage (Oyagbemi et al. 2016; Chen et al. 2007). Consequently, mitochondrial dysfunction activates intrinsic apoptotic signaling through cytochrome c release and caspase-3 activation, contributing to granulosa cell (GC) death and follicular depletion (Goud et al. 2014; Liu et al. 2016).

### Involvement of oxidative stress in cyclophosphamide-induced ovarian injury

Oxidative stress is a pathological condition characterized by an imbalance between the production of ROS and the ability of the body’s antioxidant defense system to neutralize these harmful species. This disruption in redox homeostasis results in cellular damage, including lipid peroxidation, DNA damage, and inflammation (Ali et al. 2024). CP induces oxidative stress in the ovary by generating ROS and impairing the antioxidant defense mechanism. Elevated levels of malondialdehyde (MDA), a marker of lipid peroxidation, have been observed following CP exposure (Chen et al. 2024a). CP also suppresses key antioxidant enzymes such as catalase, superoxide dismutase (SOD), and glutathione (GSH), thereby exacerbating ROS accumulation and mitochondrial dysfunction (Doğan et al. 2015; Nafees et al. 2015). Additionally, downregulation of the Nrf2 signaling pathway further impairs redox homeostasis (Ngo and Duennwald 2022). Collectively, these changes contribute to oxidative DNA damage, lipid peroxidation, and follicular apoptosis (Trujillo et al. 2023; Barberino et al. 2023). In the context of breast cancer, both ellagic acid and curcumin, natural polyphenolic compounds, have been highlighted for their role in regulating oxidative stress to restore cellular equilibrium and protect against oxidative-stress-induced cell injury (Golmohammadi et al. 2023; Golmohammadi et al. (2024).

### Involvement of inflammation in cyclophosphamide-induced ovarian injury

CP promotes ovarian inflammation by upregulating key inflammatory mediators, including NF-κB, tumor necrosis factor alpha (TNF-α), interleukin-1 beta (IL-1β), interleukin-6 (IL-6), and cyclooxygenase-2 (COX-2), all of which are closely linked to ovarian dysfunction (Gupta et al. 2010). Ovarian injury caused by CP leads to the release of damage-associated molecular patterns (DAMPs), which activate pattern recognition receptors (PRRs) such as Toll-like receptors, NOD-like receptors, and others on immune and non-immune cells (Ma et al. 2024). Activation of TLR4 triggers the NF-κB signaling pathway, resulting in increased expression of proinflammatory cytokines like IL-6 and TNF-α (Ding et al. 2016; Makled et al. 2016). While DAMP–PRR interactions serve a protective function, their excessive activation in response to CP exacerbates inflammation and contributes to ovarian damage (Ma et al. 2024).

### Involvement of apoptosis in cyclophosphamide-induced ovarian injury

**Fig. 2:**
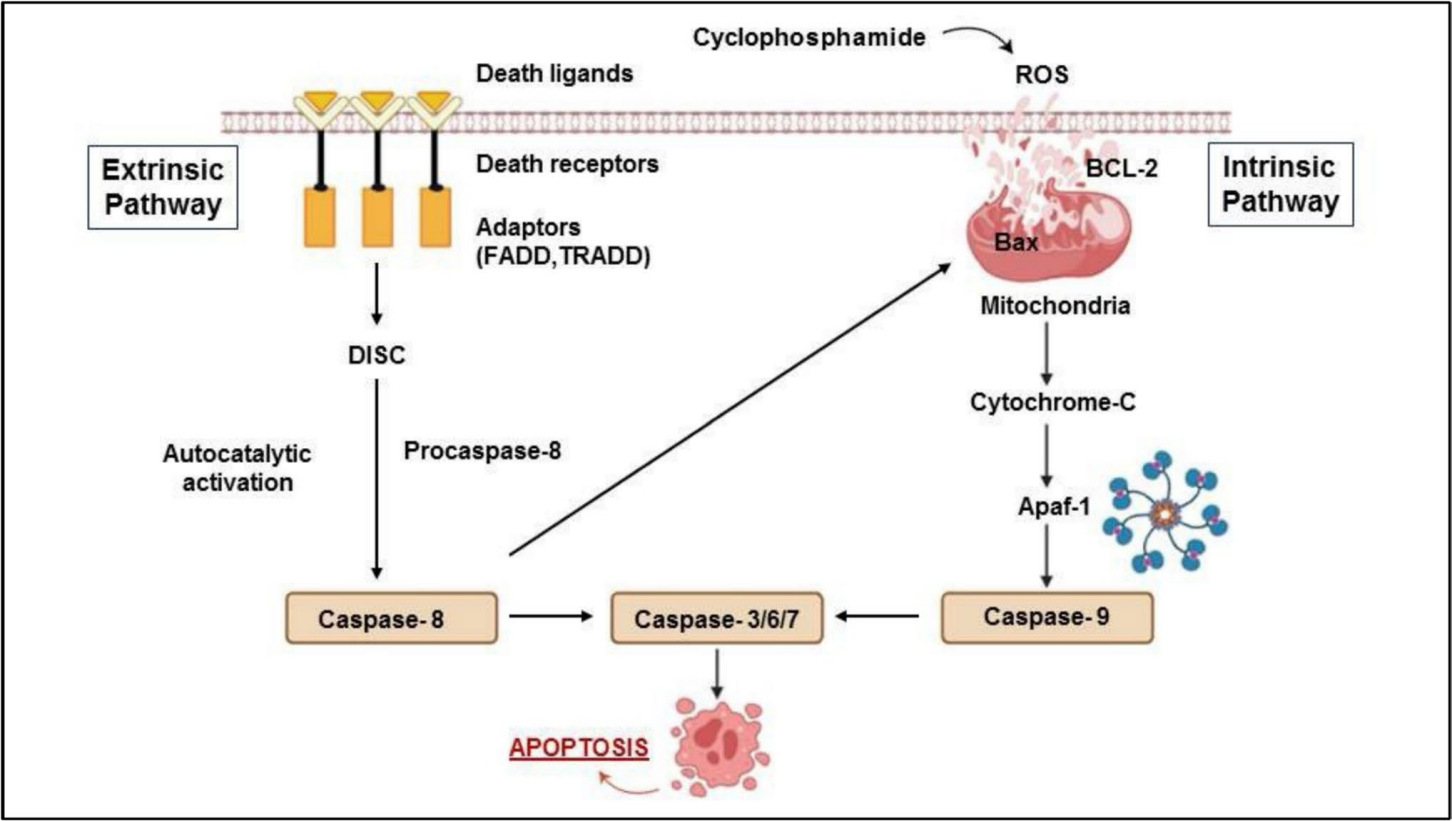
Extrinsic and intrinsic pathways of apoptosis. This figure illustrates the two main pathways of apoptosis. The extrinsic pathway (left) is initiated by death ligands binding to death receptors, leading to the formation of DISC and subsequent caspase-3 activation. The intrinsic pathway (right) involves mitochondrial dysfunction triggered by cellular stress, resulting in cytochrome c release, apoptosome formation, and caspase-3 cascade activation

Apoptosis is a key mechanism underlying CP-induced ovarian follicle depletion and contributes significantly to POF (Hassan et al. 2014; Xie et al. 2024). CP triggers both the intrinsic (mitochondrial) and extrinsic (death receptor-mediated) apoptotic pathways, primarily through oxidative stress and inflammatory signaling (Elmore 2007). The toxic CP metabolite acrolein promotes excessive ROS production, which damages mitochondrial membranes, induces lipid peroxidation, and impairs DNA integrity. This activates the intrinsic pathway via mitochondrial outer membrane permeabilization, releasing cytochrome C and forming the apoptosome complex with apoptotic protease activating factor-1 (Apaf-1) and procaspase-9 (Hashemi et al. 2004; Ghavami et al. 2004). Caspase-9 then activates caspase-3, leading to DNA fragmentation and cell death. These effects are accompanied by increased expression of pro-apoptotic proteins Bcl-2-associated X protein (Bax), p53, and decreased levels of anti-apoptotic B-cell lymphoma 2 protein (Bcl-2) (Los et al. 1995; Ghobrial et al. 2005; Yuan and Akey 2013; Jin and El-Deiry 2005). In parallel, CP-induced cytokines such as TNF-α and Fas ligand engage their receptors, forming the death-inducing signaling complex (DISC) and activating caspase-8 (Jin and El-Deiry 2005; Guicciardi and Gores 2009). This initiates the extrinsic pathway and further amplifies mitochondrial damage through BH3 interacting domain death agonist (BID) cleavage, linking both pathways at the mitochondrial level (Stergiou and Hengartner 2004). CP-evoked apoptosis via both intrinsic and extrinsic pathways is illustrated in Fig. 2.

### Involvement of TLR4 and NF-κB signaling pathway in cyclophosphamide-induced ovarian injury

**Fig. 3:**
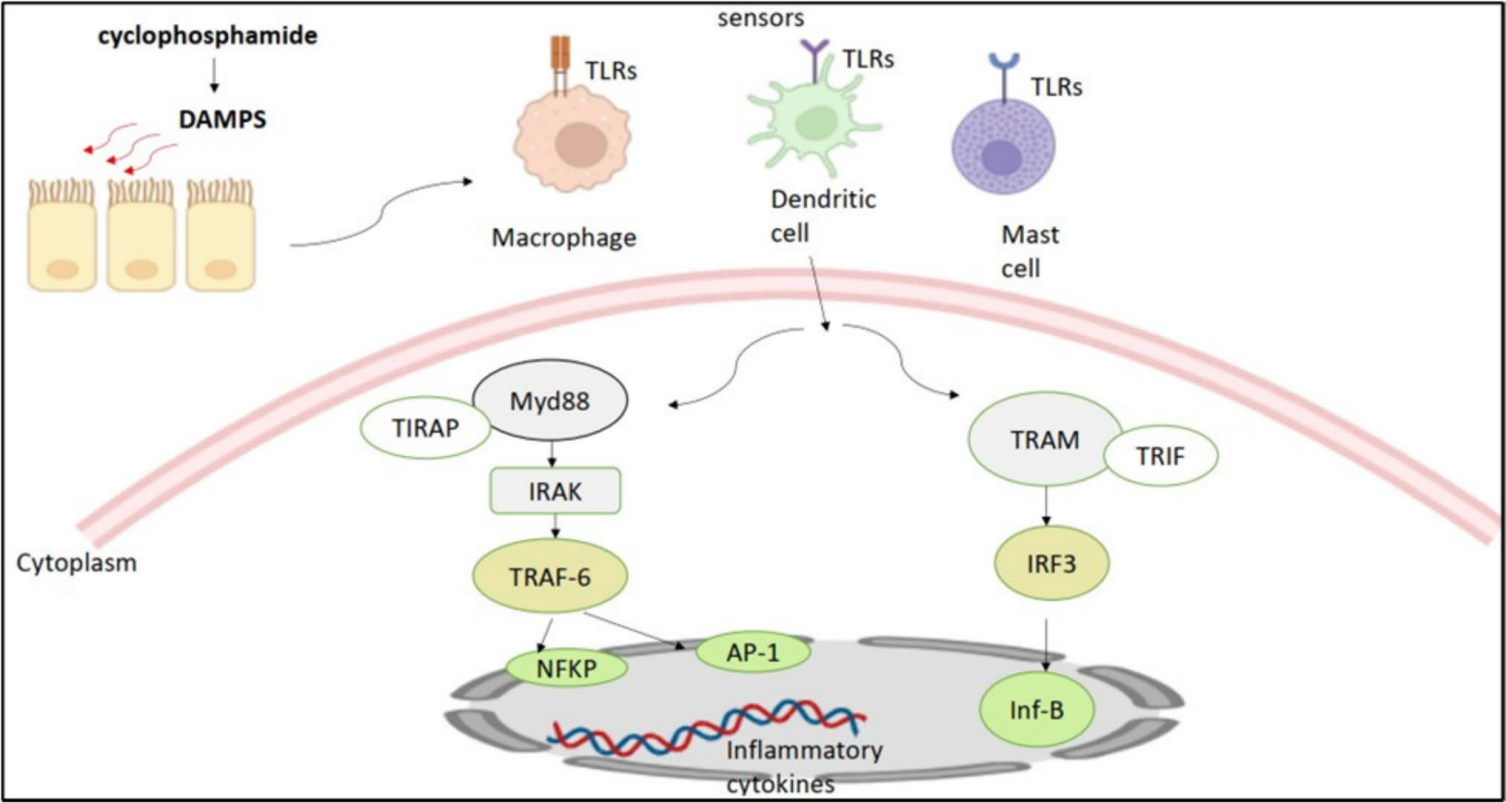
The assembly and activation of the TLR4 pathway. This figure depicts the innate immune response to DAMPs released from injured cells due to cyclophosphamide. DAMPs are recognized by TLRs on macrophages, dendritic cells, and mast cells. TLR activation triggers downstream signaling cascades involving adaptor proteins (MyD88, TIRAP, TRAM, TRIF) and kinases (IRAK), leading to activation of TRAF-6 and IRF3. These transcription factors, along with NF-κB, AP-1, and interferon regulatory factors, translocate to the nucleus to induce expression of inflammatory cytokines

It is generally agreed that TLRs, which belong to the category of transmembrane pattern recognition receptors, are essential components of the innate immune system. The majority of their expression can be found on a variety of innate immune cells, including mast cells, macrophages, and dendritic cells of the immune system (Nardo 2015; Kawasaki and Kawai 2014). According to the findings of several studies, the activation of TLRs has a significant impact on neuroinflammation and behavioral abnormalities that are brought on by chemotherapeutic medicine (Squillace and Salvemini 2022; Vichaya et al. 2015). There is a correlation between TLR4 activation and CP-induced neurotoxicity, as well as the following surplus output of pro-inflammatory cytokines and their important role in the pathophysiology of the cognitive impairment that is associated with this condition, according to a study that was conducted not too long ago (Ren et al. 2019). TLR signal transduction transpires through two primary channels: the myeloid differentiation primary response 88 (MYD-88)-independent and MYD-88-dependent pathways, which engage a consortium of adaptor proteins that facilitate the propagation of activation signals and amplify pro-inflammatory responses (Ashayeri Ahmadabad et al. 2021). TLR connects with MyD88 at the toll-interleukin 1 receptor (TIR) domain-containing adaptor protein (TIRAP), which is one of the steps in the MyD88-dependent pathway that leads to the recruitment of IL-1 receptor-associated kinase (IRAK) (Deguine and Barton 2014). In response to its activation, IRAK is responsible for activating TNF receptor-associated factor 6 (TRAF6). At some point in time, these signaling cascades will eventually phosphorylate and promote the IKK complex. This complex will then phosphorylate IκB, making it susceptible to degradation by proteasomes, resulting in nuclear translocation of NF-κB, which in turn initiates the transcription of many genes that promote inflammation. Additionally, TRAF6 can activate mitogen-activated protein kinases of the MaPK family. MaPKs are responsible for activating several transcription factors, one of which is activator protein 1 (AP-1), which in turn causes the synthesis of a great deal of inflammatory mediators (Kawasaki and Kawai 2014; Hou et al. 2017; Walsh et al. 2015). On the other hand, in the pathway that is not dependent on MyD88, TLR is responsible for recruiting adaptor proteins such as translocating chain-associated membrane protein (TRAM) and TIR-domain-containing adaptor-inducing interferon (TRIF). Interferon regulatory factor 3, also known as IRF3, is activated when these two proteins go through the process of dimerization, which ultimately results in the release and production of interferon beta, also known as IFN-γ (Duan et al. 2022; Ullah et al. 2016). NF-κB is the primary transcription factor that is responsible for regulating the synthesis of several genes that are associated with inflammation. Several studies have demonstrated that the occurrence of CP-induced ovarian injury is closely associated with the intensification of inflammatory responses. This intensification is facilitated by the upregulation of TLR4/NF-κB expression, which in turn leads to the overproduction of inflammatory mediators. These mediators include TNFα, nitric oxide (NO), IL-1β, and IL-6 (Khallaf et al. 2023; Ran 2015). The activation of the pathway is illustrated in Fig. 3.

### Role of NLRP3 inflammasome/caspase 1 signaling pathway in cyclophosphamide-induced ovarian injury

**Fig. 4:**
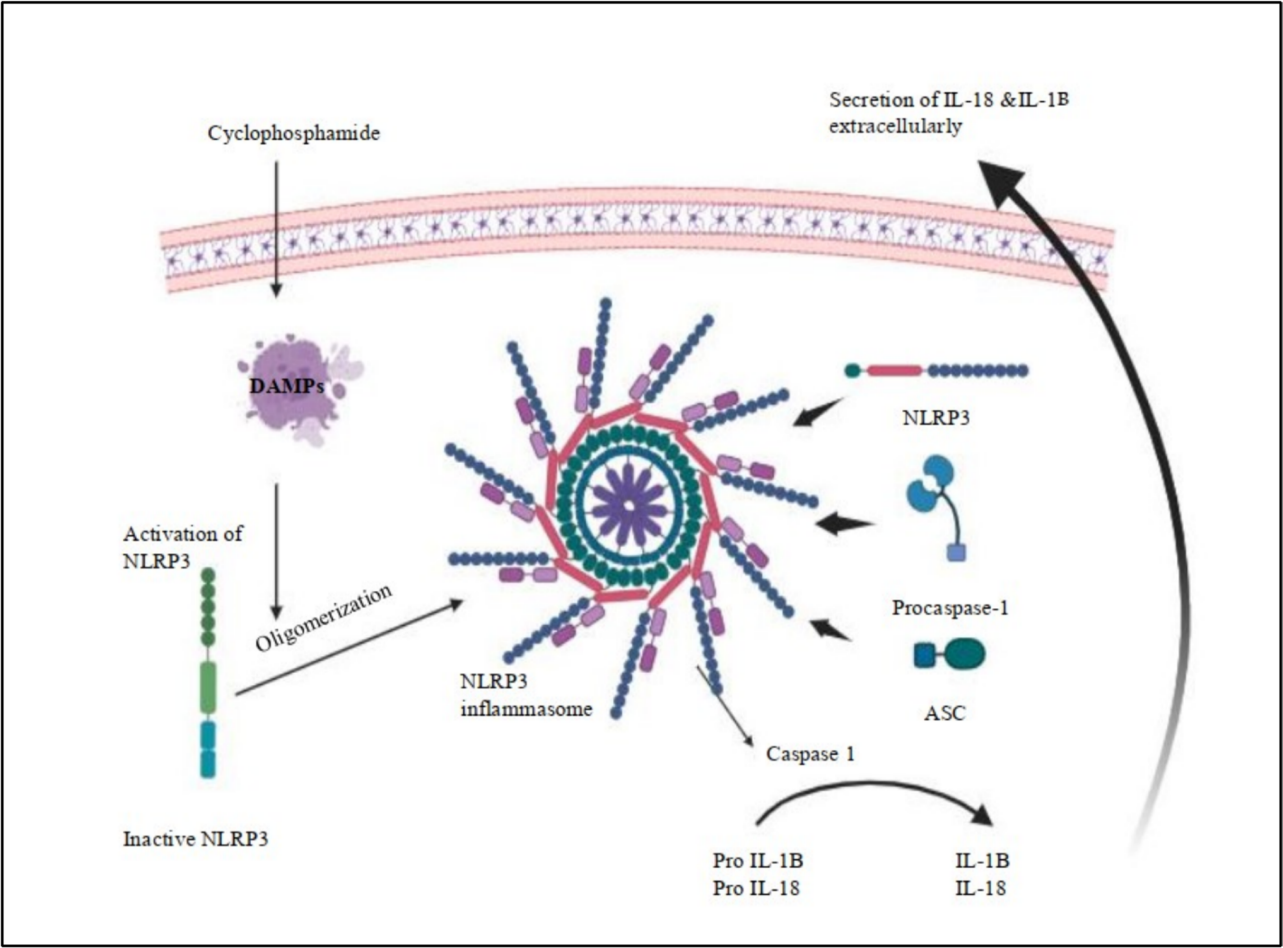
The assembly of the NLRP3/caspase-1/GSDMD pathway. This figure illustrates the NLRP3 inflammasome activation pathway triggered by cyclophosphamide. DAMPs activate the NLRP3 inflammasome complex. The inactive NLRP3 protein oligomerizes to form the inflammasome that recruits the ASC and procaspase-1. This assembly leads to caspase-1 activation, which cleaves the inactive precursors pro-IL-1β and pro-IL-18 into their mature forms

Inflammasomes, which are multiprotein complexes, assemble in the cytoplasm and are triggered by many endogenous and exogenous stimuli, including ROS and DAMPs. It has been established that inflammasomes comprise many subtypes (Blevins et al. 2022a; Dai et al. 2020). The most prominent among them is the NLRP3 inflammasome. Upon stimulation, procaspase 1, NLRP3, and apoptosis-associated speck-like protein (ASC) converge to form the NLRP3 inflammasome (Zheng et al. 2020a; Blevins et al. 2022b). In subsequent steps, the activation of pro-caspase 1 results in the formation of active caspase 1, which in turn degrades pro-IL-1β and pro-IL-18 into their mature and dynamic forms. This causes an increase in the production of additional inflammatory cytokines, which in turn causes the inflammatory responses to become more intense (Kelley et al. 2019; Abdelnaser et al. 2025a). It has been found that ovarian failure is related to an aggressive inflammatory response. Pyroptosis is characterized by the production of proinflammatory intracellular agents, such as IL-18 and IL-1β, in addition to the creation of pores in the plasma membrane, cellular swelling, and membrane rupture that are induced by the gasdermin family. Gasdermin D (GSDMD) is cleaved by activated caspase-1, which results in the formation of membrane holes that promote pyroptosis (Liu et al. 2018a; Shi et al. 2015). The transcription factor that ultimately increases the synthesis of proinflammatory proteins, including pro-IL-1β, pro-IL-18, NLRP3, and caspase-1, is mostly governed by NF-κB, which provides a significant amount of overall control (Shi et al. 2015; Du et al. 2020). CP-induced ovarian damage is highly connected with this pathway, as revealed by several investigations (Zhang et al. 2021; Vindevogel et al. 2016; Navarro-Pando et al. 2021). The assembly of the NLRP3 inflammasome is shown in Fig. 4.

### Role of SIRT1 in cyclophosphamide-induced ovarian injury

SIRT1 plays a significant part in preserving the function of ovarian tissue and reducing the negative effects of ovarian aging (Li et al. 2023b). Several investigations have established SIRT1’s regulatory role within the granulosa cells (Rofaeil et al. 2024). Zhao and colleagues subsequently identified that SIRT1 is involved in regulating processes associated with follicular atresia through GC apoptosis in porcine ovaries exhibiting follicular atresia (Zhao et al. 2014). Through the activation of SIRT1, Nie et al. were able to successfully ameliorate the effects of CP-induced POF in a mouse ovarian model. This was accomplished by significantly lowering the expression of the pro-apoptotic protein Bax and increasing the production of the anti-apoptotic protein Bcl-2 (Wang and Li 2021). Han et al. found that SIRT1 improved the resilience of granulosa cells to apoptosis (Han et al. 2017). Similar findings were discovered by Sirotkin in swine ovarian granulosa cells. In these cells, SIRT1 has been demonstrated to influence the transcription factors p53 and NF-κB, both of which are involved in the modulation of GC apoptosis and proliferation (Sirotkin et al. 2014). The synthesis of the SIRT1 enzyme in ovarian cells is markedly diminished in CP-induced ovarian damage, which exacerbates inflammatory responses (Li et al. 2023b; Chen et al. 2024b). The mechanism by which SIRT1 hinders the activity of several redox-sensitive pro-inflammatory mediators, such as NF-kB and NLRP3, can shed light on this phenomenon. SIRT1 can restrict the transcriptional activity of NF-κB through the process of deacetylation of the p65 subunit. This may make it easier for the NF-kB complex to interact with IκB, which will then cause the NF-kB complex to move from the nucleus to the cytoplasm, reducing the expression of genes that are associated with inflammation (Gregorio et al. 2020).

### Role of Nrf2/Keap1 pathway in cyclophosphamide-induced ovarian injury

Nrf2 is the primary regulator of cellular responses to external stimuli (Kobayashi et al. 2004). Both antioxidants and detoxifying enzymes are encoded by the Nrf2, which makes it possible for a redox-sensing system to function (Alaaeldin et al. 2024; Mohyeldin et al. 2025a). By causing its activity to be adversely modulated by proteasomal degradation, Kelch-like ECH-associated protein 1 (KEAP1) acts as a natural inhibitor of the natural regulatory factor Nrf2 (Wang et al. 2008). In response to the presence of xenobiotics, the Nrf2/Keap1 pathway is activated, which leads to the release of Nrf2, which is accomplished by translocating to the nucleus. After that, it can affix itself to the sequences of the antioxidant response element, which are responsible for regulating several particular genes, such as glutathione S-transferase and HO-1 (Taguchi et al. 2011). Nrf2 is a transcription factor that targets genes that encode enzymes involved in the metabolism of drugs, transporters, antioxidant enzymes, and enzymes involved in the metabolism of heme and iron. Through the reduction of cell death and the enhancement of the cellular redox state, hyperactivation of Nrf2 was able to minimize oxidative stress (Suzuki et al. 2013; Mohyeldin et al. 2025b). Several studies conducted in the past have shown that ovarian damage caused by CP is associated with a decrease in the expression of Nrf2 (Li et al. 2024; El-Marasy et al. 2025; Chen et al. 2021).

### Role of α-klotho in cyclophosphamide-induced ovarian injury

Those individuals who have been diagnosed with POF have a notable decrease in the expression of α-Klotho, which suggests a solid connection between decreased levels of α-Klotho and the onset of ovarian aging (Xie et al. 2021). Klotho expression was dramatically reduced in animal models of CP-induced POF compared to the control group (Liu et al. 2019a). Prior research revealed that rats intoxicated with CP had a marked decrease in ovarian α-Klotho, while the elevation of α-Klotho levels alleviated the ovarian damage induced by CP (Biyik et al. 2021; Khallaf et al. 2025; Rofaeil et al. 2025). The pathological function of α-Klotho in POF remains inadequately elucidated; nevertheless, it can be interpreted by viewing POF as a pathological aging phenomenon, given the depletion of ovarian reserve during POF. An earlier study has shown that α-Klotho plays a significant role in the development of oocytes by aiding maturation through the activation of wingless-related integration site (Wnt) signaling pathways (Kim et al. 2020, 2023) as well as controlling the development of ovarian folliculogenesis through the phosphoinositide 3-kinase (PI3K)/protein kinase B (AKT)/mammalian target of rapamycin (mTOR) pathway (Hu et al. 2022). The decrease in ovarian α-Klotho expression led to a decrease in autophagy, which negatively impacted the cells’ ability to eliminate ROS. This, in turn, disrupted the normal cellular activity and triggered apoptosis, which ultimately led to a loss of ovarian reserve (Liu et al. 2019b; Sachs-Guedj et al. 2024). Furthermore, recent research has provided evidence to support the idea that the activation of α-Klotho inhibited the TLR4 signaling pathway (Typiak and Piwkowska 2021), apoptosis (Sugiura et al. 2005), and oxidative stress (Oh et al. 2015; Abdelnaser et al. 2025b).

### Role of the PTEN\PI3K\AKT pathway in cyclophosphamide-induced ovarian injury

In both healthy and pathological conditions, the PI3K/AKT/mTOR signaling pathways are crucial for several aspects of cellular development and survival (Porta et al. 2014). PI3K is responsible for facilitating the phosphorylation of phosphatidylinositol, which in turn regulates processes such as cell motility, survival, differentiation, growth, and intracellular transport (Cully et al. 2006). The term “protein kinase B” (PKB) or “AKT” refers to a group of three serine/threonine-specific protein kinases that may be involved in a variety of cellular processes, such as apoptosis, proliferation, transcription, and migration (Revathidevi and Munirajan 2019; Mohyeldin et al. 2024). The PI3K/AKT pathway generates intracellular signaling cascades and comprises many signaling molecules, including kinases, phosphatases, and transcription factors. This prominent intracellular signaling pathway also contributes to the stimulation of primordial follicles in the ovary (Cantley 2002). The AKT kinase, which plays a significant role in the activation of primordial follicles, exerts both direct and indirect effects on the activation of follicles through a wide variety of substrates that are present in human ovarian granulosa cells and oocytes (Cecconi et al. 2012). Among the many AKT substrates, the forkhead box O3 (Foxo3) protein was initially recognized as a regulator of the primordial follicle activation pathway. This discovery was made available to researchers. Experiments have shown that the expression of the active Foxo3 gene in mouse oocytes causes a delay in the development of oocytes and follicles. This is because all dormant follicles in the pubertal ovary are triggered prematurely in animals that are lacking in the Foxo3 gene. This suggests that the normal expression of the Foxo3 gene can prevent the formation of follicles and keep follicles in a dormant condition (Liu et al. 2007). An investigation conducted by Goldbraikh and colleagues revealed that the PI3K/AKT/Foxo3 signaling pathway is the primary mechanism responsible for controlling growth and metabolism in every cell (Goldbraikh et al. 2020). The action of the phosphatase and tensin homolog gene PTEN, which inhibits the PI3K/AKT pathway, causes primordial follicles to remain dormant for a significant period throughout the process of follicle development. After PTEN inhibition has been removed from primordial follicles, PI3K activation takes place in these cells. This ultimately resulted in the transition of phosphatidylinositol 4,5-bisphosphate into phosphatidylinositol-3, 4, 5-triphosphate, which in turn activated phosphoinositide dependent protein kinase-1 and stimulated AKT. Primordial follicles are activated when the Foxo3 protein, which is located far downstream, is phosphorylated. This causes the Foxo3 protein to lose its ability to perform transcriptional functions and causes it to move from the nucleus to the cytoplasm, where it is then targeted for degradation (John et al. 2008). As a result of its role as a negative regulator of PI3K, PTEN can block the PI3K signaling pathway. It has been demonstrated through research that the activation of latent primordial follicles occurs when the expression of the PTEN gene is suppressed in mice because of increased activity in the PI3K signaling pathway. By removing the PTEN gene from the oocytes of mouse primordial follicles, it is possible that the formation of primordial follicles and the early activation of the entire primordial follicle pool during puberty would be facilitated. Not only does this cause follicular depletion in early adulthood mice, but it also occurs in POF in mice (Adhikari et al. 2012). Animal studies indicate that the PTEN/PI3K/AKT signaling pathway is mostly responsible for maintaining follicular dormancy or facilitating follicle activation (2010). The ovarian follicular reserve is swiftly diminished and prematurely activated in genetically modified mouse models when one or more components of this pathway are deliberately eliminated in the oocyte (Castrillon et al. 2003). Short-term activation of PI3K or suppression of PTEN in human cortical tissue facilitates follicle development and results in the depletion of primordial follicles (Li et al. 2010). CP influences the PI3K/AKT pathway in two distinct ways. Overactivation of PI3K/AKT induces premature activation of the dormant follicle pool and follicular burnout, culminating in fast depletion and eventual POF, whereas inhibition of PI3K/AKT may transpire in developing follicles, resulting in GC death and follicular atresia (Zhang et al. 2018; Zhou et al. 2017).

### Interconnected molecular pathways underlying cyclophosphamide-induced ovarian injury

CP-induced POF involves a highly interconnected molecular cascade, where oxidative stress acts as the central initiator. The metabolic byproducts of CP, particularly acrolein, generate excessive ROS, disrupting redox balance and damaging ovarian cellular components (Nie et al. 2021). This oxidative burden serves as a trigger for innate immune signaling, particularly through the activation of TLR4, which subsequently activates the NF-κB pathway. NF-κB translocates to the nucleus and drives the expression of pro-inflammatory cytokines such as TNF-α, IL-1β, and IL-6, which not only propagate inflammation but also contribute to the activation of the NLRP3 inflammasome (Zhang et al. 2021). The NLRP3 complex promotes the cleavage of pro-caspase-1 into active caspase-1, facilitating the maturation and release of IL-1β and IL-18, amplifying inflammation and pyroptosis within the ovarian microenvironment (Yin et al. 2023). Parallel to this, sustained oxidative and inflammatory stress destabilizes mitochondrial integrity, activating the intrinsic apoptotic pathway. This leads to the release of cytochrome c and subsequent activation of caspase-9 and executioner caspase-3, resulting in GC apoptosis and follicular depletion. Under physiological conditions, the Nrf2 pathway acts as a primary defense mechanism by inducing antioxidant enzymes such as HO-1, SOD, and catalase. However, CP suppresses Nrf2 signaling, weakening the cellular defense mechanism. SIRT1, a redox-sensitive NAD⁺-dependent deacetylase, normally exerts cytoprotective effects by inhibiting NF-κB, promoting Nrf2 activation and maintaining mitochondrial function; its downregulation under the effect of CP further impairs these protective responses (El-Marasy et al. 2025). Additionally, α-Klotho, an anti-aging protein with known antioxidant and anti-apoptotic functions, is reduced by CP exposure. This reduction contributes to heightened oxidative damage, inflammation, and apoptotic sensitivity. α-Klotho also positively influences SIRT1 and Nrf2 signaling, suggesting a feedback loop wherein its depletion accelerates ovarian injury (Rofaeil et al. 2025). Altogether, these pathways form a tightly integrated network, where oxidative stress, inflammatory signaling, and apoptosis reinforce one another, while regulatory pathways such as Nrf2/HO-1, SIRT1, and α-Klotho are suppressed, shifting the cellular balance toward damage and follicular loss.

## Therapeutic protection against cyclophosphamide-induced ovarian injury in experimental studies

### Buspirone

Buspirone (BUS) is a medication frequently prescribed for anxiety and depression, Functioning as a partial agonist at 5-HT₁A receptors and an antagonist at dopamine D₂ receptors through presynaptic modulation of serotonin release (Loane and Politis 2012; Abdel-Salam et al. 2017). It has also been used adjunctively to manage chemotherapy-induced dyspnea and emesis in patients treated with CP (Alfieri and Cubeddu 1995; Wolff and Leander 1997; Peoples et al. 2016). Additionally, BUS improved gastric accommodation and relieved gastroparesis conditions, often associated with chemotherapy-related neuropathy or tumor burden (Parkman et al. 2023; Sayuk 2023). Beyond its gastrointestinal effects, BUS has shown potential in mitigating vasomotor and sexual symptoms such as hot flashes and hypoactive sexual desire, which are commonly observed in women with POF (Shumilov and Touitou 2010; Croft 2017). Recent studies demonstrated that BUS exerts anti-inflammatory and antioxidant actions via multiple pathways. It downregulates NLRP3 inflammasome activity, suppresses the TLR4/NF-κB axis, and activates the Nrf2/HO-1 signaling cascade, offering neuroprotection and reducing inflammation in various animal models (Althagafy et al. 2023; Rashidian et al. 2022). BUS also modulates apoptosis by influencing the Bax/Bcl-2/caspase-3 pathway (Sharifi et al. 2015) and supports metabolic regulation via AMP-activated protein kinase (AMPK) pathway activation (Lee et al. 2023). In CP-induced POF, BUS has been shown to mitigate oxidative stress, inflammation, and apoptosis. These effects are mediated through inhibition of the NF-κB/NLRP3/caspase-1 and Bax/Bcl-2/caspase-3 pathways, alongside activation of Nrf2/HO-1, p-AMPK, and α-Klotho signaling (Khallaf et al. 2025). Its established side effects in the clinical setting include dizziness, headache, restlessness, nausea, and, less commonly, serotonin syndrome when combined with other serotonergic drugs (Loane and Politis 2012). Importantly, there is a lack of direct clinical data regarding long-term reproductive safety in humans.

### Levomilnacipran

Levomilnacipran (LVM) is a selective serotonin and norepinephrine reuptake inhibitor (SNRI) approved by the FDA in 2013 for the treatment of major depressive disorder (Fanelli et al. 2021; Sharata et al. 2025). To enhance functional recovery in individuals who have suffered an ischemic stroke, LVM is currently undergoing development and has advanced to a phase II clinical trial (Hair et al. 2013a). It exhibits analgesic effectiveness and mitigates weariness linked to depression (Hair et al. 2013a, 2013b). Its favorable safety profile and low discontinuation rate contribute to strong patient adherence (Asnis and Henderson 2015; Montgomery et al. 2013). LVM is also used to treat fibromyalgia, neuropathic pain, and burning mouth syndrome, which commonly affect female cancer patients undergoing chemotherapy (Deardorff and Grossberg 2014; Saraceni et al. 2014; Bernstein et al. 2013; Ohnami et al. 2012). Additionally, women with cancer or fibromyalgia often experience comorbid depression and hot flashes, symptoms for which SNRIs have demonstrated benefit (Thiagarajah et al. 2014; Yepez et al. 2022; Raison and Miller 2003). Recent evidence showed that LVM exerts neuroprotective and anti-inflammatory effects by suppressing the TLR4/p38 MAPK/NF-κB and Bax/Bcl-2/caspase-3 pathways (Wu et al. 2024; Li et al. 2023c). In a rat model of CP-induced POF, LVM significantly reduced ovarian oxidative damage, inflammation, and apoptosis via suppression of the TLR4/p38 MAPK/NF-κB and Bax/Bcl-2/caspase-3 pathways, in addition to the activation of the α-Klotho protein pathway (Rofaeil et al. 2025). It is primarily associated with side effects such as nausea, increased heart rate, hyperhidrosis, constipation, urinary hesitation, sexual dysfunction, and potential increases in blood pressure (Asnis and Henderson 2015).

### Cilostazol

Cilostazol is a derivative of the 2-oxo-quinoline system that possesses antithrombotic, vasodilatory, antimitogenic, and cardiotonic properties. This compound is an extremely effective inhibitor of phosphodiesterase-3A (Abdel-Aziz et al. 2020). Cilostazol has been shown to have considerable antithrombotic effects in vivo, as well as to inhibit the aggregation of platelets (Minami et al. 1997). Cilostazol can effectively lower serum triglyceride levels while simultaneously causing a little elevation in HDL cholesterol levels (Elam et al. 1998). Cilostazol significantly reduced ovarian tissue oxidative stress markers in rats treated with CP via decreasing MDA levels and increasing SOD and GSH levels compared to control rats (Abdel-Aziz et al. 2020). Cilostazol elevates intracellular cyclic nucleotides, and increased amounts of these nucleotides have been demonstrated to diminish ROS production and cellular dysfunction. In the comparison between the treated group and the CP group, a notable enhancement in the ovarian gene expression of HO-1 and Nrf2 was observed. Consequently, the antioxidant and anti-inflammatory effects of cilostazol in CP-associated ovarian toxicity are related to HO-1 induction (Abdel-Aziz et al. 2020). Cilostazol inhibited ovarian apoptosis and alleviated CP-induced ovarian destruction by upregulating HO-1 and cyclic adenosine monophosphate. It can cause headache, palpitations, diarrhea, dizziness, and edema. In patients with heart failure or arrhythmias, cilostazol is generally contraindicated due to the risk of increased cardiac events (Kherallah et al. 2022).

### Diosmin

Diosmin is a recognized natural flavonoid utilized in the treatment of varicose veins and chronic venous insufficiency (Zheng et al. 2020b). Recent investigations have demonstrated that diosmin possesses a broad spectrum of pharmacological activities, including anti-inflammatory effects (Berköz 2019), antioxidant (Srinivasan and Pari 2012), anti-diabetic (Hsu et al. 2017), anti-cancer (Naso et al. 2016), and retinal protection properties (Tong et al. 2013). An earlier preclinical study demonstrated the effectiveness of diosmin against CP-induced POF. The study included hormonal evaluations of FSH, estradiol (E2), and AMH, as well as histopathological examinations of ovarian tissues, evaluations of oxidative stress levels, and measurements of the relative expression of microRNA-145 in conjunction with its target genes, vascular endothelial growth factor B (VEGF-B) and regulator of cell cycle (RGC32). Oxidative stress indicators, as well as levels of AMH and E2, were all improved by diosmin therapy. At both low and high dosages of diosmin, the histological changes were greatly alleviated. The level of expression of miRNA-145 was shown to be enhanced following the administration of a high dose of diosmin. The administration of diosmin resulted in a significant decrease in the number of atretic follicles and an increase in the total count of developing follicles at various stages of folliculogenesis within the ovarian cortices. The results were dependent on the dosage that was administered (Abogresha et al. 2021). Being a natural flavonoid, diosmin’s side effects are generally mild, such as gastrointestinal upset, headache, or mild skin reactions. Allergic reactions are rare (Gerges et al. 2022). Long-term reproductive safety data are scarce, as usage is mostly extrapolated from its vascular applications.

### Donepezil

Donepezil is a selective acetylcholinesterase inhibitor commonly administered for individuals with mild to moderate Alzheimer’s disease (Rogers and Friedhoff 1996; Rogers S, Farlow M, Doody R, Mohs R, Friedhoff L, Group* DS 1998; Burns et al. 1999). Donepezil has also demonstrated efficacy for persons at both extremes of the Alzheimer’s disease spectrum: benign, incipient conditions (Seltzer et al. 2004) and those with moderate-to-severe impairment (Feldman H, Gauthier S, Hecker J, Vellas B, Subbiah P, Whalen E, Group* DMSI 2001), including patients in nursing homes (Tariot et al. 2001). Moreover, cholinesterase inhibitors like donepezil may prove beneficial in vascular dementia (Black et al. 2003; Wilkinson D, Doody R, Helme R, Taubman K, Mintzer J, Kertesz A, Pratt R, Group* DS 2003) and dementia associated with Parkinson’s disease (Leroi et al. 2004; Aarsland et al. 2002). In the experimental model of CP-induced POF, donepezil was found to increase serum AMH levels in a dose-dependent manner. A decrease in the expression of ovarian TLR4, NLRP3, IL-6, and TNF-α was observed when donepezil was administered. Furthermore, donepezil restored all histopathological aberrations caused by CP. This was demonstrated by the presence of typically growing follicles in the early stages of maturation and a reduced number of atretic follicles. An increase in the dose of donepezil led to an improvement in the protection, as demonstrated by the presence of healthier follicles and a decrease in the number of atretic follicles. The protective mechanism of donepezil was achieved by inhibiting the production of NO, proinflammatory cytokines, the TLR-4/NF-κB/NLRP3 inflammasome pathway, and apoptosis (Zidan et al. 2024). Donepezil causes gastrointestinal disturbances (nausea, vomiting, diarrhea), muscle cramps, insomnia, bradycardia, syncope, and (rarely) cardiac conduction abnormalities, particularly in the elderly or those with underlying heart disease (Jackson et al. 2004).

### LCZ696

In July 2015, the FDA authorized LCZ696, a pioneering medicine that integrates sacubitril, a neprilysin enzyme inhibitor, and valsartan, an angiotensin II receptor AT1 antagonist, for the treatment of heart failure and perhaps hypertension (McMurray et al. 2014; Lm 2010; Hubers and Brown 2016). LCZ696 has shown the capacity to reduce oxidative stress and inflammation by increasing endogenous vasoactive peptide levels through neprilysin inhibition (Mohyeldin et al. 2023). This is in addition to the benefits that it offers for the cardiovascular system (Jing et al. 2017). LCZ696 improved endothelial function through the inhibition of the TLR4/NF-κB signaling pathway. LCZ696 was able to reduce inflammatory responses, decrease the NLRP3 inflammasome, and offer protection against myocardial infarction and early diabetic nephropathy (Khallaf et al. 2023; Shen et al. 2021; Li et al. 2020; Pan et al. 2022; Gao et al. 2021). Through its antioxidant, anti-inflammatory, and anti-apoptotic activities, LCZ696 demonstrated a protective effect against CP-induced ovarian damage in rats. This protective effect can be interpreted based on two complementary suggested pathways, initially, through the direct inhibition of the NLRP3/caspase-1/GSDMD C-NT signaling pathway. Furthermore, inhibiting the signaling pathway of TLR4, MYD88, and NF-B P65 ultimately resulted in a decrease in NLRP3, pro-IL-1β, pro-IL-18, and TNF-α (Khallaf et al. 2023). It is mainly associated with hypotension, hyperkalemia, renal impairment, and, less commonly, angioedema. It is contraindicated in pregnancy due to teratogenic effects and potential fetal toxicity (Chua et al. 2021).

### Melatonin

The pineal gland is the principal producer of the indoleamine hormone melatonin, which aids in the regulation of sleep–wake cycles and circadian rhythms (Stehle et al. 1991). In addition to its chronobiological function, melatonin has several other beneficial impacts on different organs, such as the reproductive system, including reducing inflammation, preventing cell death, and protecting cells from free radicals (Reiter et al. 2016; Esposito et al. 2019; Cipolla-Neto and Amaral (2018). Melatonin is produced in several organs, including the ovaries (Jang et al. 2017). It influences cellular signaling pathways that are involved in cell survival, oxidative stress response, and immunological regulation through melatonin receptors MT1 and MT2 (Jang et al. 2017). Melatonin has emerged as a promising fertoprotective agent against CP-induced ovarian injury, with evidence from animal studies and mechanistic investigations (Abdi et al. 2024). The melatonin administration before or alongside CP treatment preserved the ovarian reserve by maintaining the number of primordial, primary, and growing follicles, reducing follicular atresia, and activating the Hippo signal pathway (Xu et al. 2022a). Melatonin helped to sustain physiological levels of AMH, E2, and inhibin B, while reducing elevated FSH and LH levels, thereby supporting ovarian endocrine function (Feng et al. 2022). It also inhibited the apoptosis of granulosa cells, which are vital for follicle survival and hormone production (Feng et al. 2022). Melatonin mitigated CP-induced oxidative stress and excessive autophagy in granulosa cells via modulating the PI3K/AKT/mTOR signaling pathway (Xu et al. 2022b; Barberino et al. 2022; Liu et al. 2022). Melatonin is well-tolerated with minor side effects such as drowsiness, headache, and dizziness. High doses may interfere with circadian rhythms, affect mood, or interact with anticoagulants (Anderson and Maes 2012).

### Moxibustion

In traditional Chinese medicine, moxibustion is the practice of lighting moxa at acupoints and in certain locations. Moxibustion protects against ulcerative colitis, post-inflammatory irritable bowel syndrome, and chronic exercise-induced fatigue (Li et al. 2019; Ma et al. 2016; Bao et al. 2019). Moxibustion has been shown to reduce ovarian damage brought on by CP by suppressing NLRP3 activation (Niu et al. 2022; Zhao et al. 2010). The activation of NLRP3 inflammasome is mostly induced by excessive ROS produced from compromised mitochondria. The age-related decrease in female fertility has been associated with the NLRP3 inflammasome, rendering this inflammatory complex a potential therapeutic target for infertility treatment. Inhibiting NLRP3 activation can mitigate reproductive aging in female rats (Navarro-Pando et al. 2021). Furthermore, it has been demonstrated that animals that have NLRP3 inflammasome activation have ovarian dysfunction as well as fibrosis (Wang et al. 2020). Through the reduction of mRNA and protein expression levels of NLRP3, ASC, GSDMD, and caspase 1, as well as the reduction of serum and ovarian levels of IL-18 and IL-1β, moxibustion therapy, when administered to a rat model of CP-induced POF, demonstrated a significant suppression of NLRP3 activation (Yin et al. 2023). Moxibustion is considered very safe; risks include burns or allergic reactions, but systemic adverse effects are rare and not observed in experimental ovarian injury models (Park et al. 2010).

### Resveratrol

Several plant species are used to extract resveratrol (RES), a naturally occurring polyphenol compound (Oh and Shahidi 2018). RES is renowned for its cytoprotective effects against various diseases, attributable to its numerous biological activities, including anti-inflammatory, anti-cancer, anti-oxidative, anti-aging, and estrogen-regulatory properties (Athar et al. 2007). For in vitro-cultured follicles and zygotes, RES has a favorable regulatory effect (Sugiyama et al. 2015). Furthermore, resveratrol is an SIRT1 activator (Howitz et al. 2003). A key player in controlling follicular growth and development is the SIRT1 signaling pathway (Zhou et al. 2015). Prior research has shown that rat granulosa cells express SIRT1 mRNA (Nie et al. 2020; Morita et al. 2012). ROS buildup can be effectively removed by RES (Park and Pezzuto 2015). RES protects against ovarian injury induced by chemotherapy by increasing the enzymatic activity of SOD and CAT, and downregulating apoptosis. RES is a potent antioxidant that efficiently eliminates lipid peroxidation and DNA damage brought on by ROS (Leonard et al. 2003). Under stressful circumstances, RES has been shown to affect cellular functions, including autophagy and the apoptotic cascade. The beneficial action of RES is largely dependent on its concentration, and it shows strong effects in reducing ovarian injury (Nie et al. 2020). This polyphenol is generally well-tolerated, but high doses can lead to gastrointestinal upset, headache, or elevated liver enzymes in rare cases (Shaito et al. 2020).

### Irbesartan

Irbesartan (IRB) is a synthetic non-peptide antagonist of angiotensin II that possesses agonistic activity for the peroxisome proliferator-activated receptor-gamma (PPAR-ɣ) (Vignier et al. 2014; Zhang et al. 2013). As PPAR-ɣ activation produces anti-inflammatory effects and an improvement in endothelial function, lipid metabolism, and a reduction in ROS production (Martin et al. 2012; Hu et al. 2014; Ibrahim et al. 2025), IRB reduced inflammatory parameters and prevented apoptotic cell death (Anjaneyulu and Chopra 2004). Since PPAR-ɣ agonistic drugs such as IRB are responsible for strong organ-protective effects, including anti-fibrotic, anti-oxidant, and anti-inflammatory effects, IRB was proven to be effective in protection from CP-induced ovarian injury (Wang et al. 2013a). As mentioned in the previous experimental study, serum FSH was increased, and serum estradiol was decreased after CP administration. Moreover, CP administration significantly increased ovarian TNF-α, MDA, myeloperoxidase (MPO), and caspase-3 levels. All previously mentioned parameters have been normalized after IRB administration. On the other hand, IL-10, GSH levels, and SOD activity significantly decreased after CP administration, which was corrected after IRB administration (Abdel-Raheem et al. 2015). The most typical side effects are hypotension, dizziness, hyperkalemia, and rare angioedema. Like all drugs in this class, they are contraindicated in pregnancy due to established teratogenic risks (Bramlage et al. 2009).

### Mirtazapine

Mirtazapine, often known as MTZ, is a medication that is approved for the treatment of serious depression (Davis and Wilde 1996). It also has antioxidant activity besides its antidepressant effect (Altuner et al. 2013). Previous studies have also stated that MTZ with various doses (15, 30, and 60 mg/kg) significantly alleviated indomethacin-induced mucosal damage (El-Awdan and Zaki 2013). Given the anticipated prevalence of depression and anxiety among cancer patients due to the detrimental effects of chemotherapy and the heightened severity observed in infertile women, MTZ pretreatment in cancer patients undergoing CP as an anti-cancer therapy would be beneficial in various respects (Altuner et al. 2013). A prior study indicated that pretreatment with MTZ significantly enhanced ovarian weight and the number of mature follicles, which markedly diminished following CP (Khedr 2015). In addition, the administration of MTZ decreased the raised levels of NO and MDA, enhanced the activity of glutathione peroxidase (GPx) and SOD, and decreased the activity of MPO (Khedr 2015). Consequently, it is posited that MTZ may augment the antioxidant activity of the ovary and reduce the formation of peroxynitrite, generated by the interaction between NO and superoxide anion. This is achieved by neutralizing the superoxide anion through the action of SOD. MTZ is prone to causing sedation, increased appetite, weight gain, dry mouth, and occasionally agranulocytosis. Caution is warranted with other serotonergic drugs (Nutt 2002).

### Sildenafil

Erectile dysfunction and pulmonary arterial hypertension are two conditions for which sildenafil, an inhibitor of phosphodiesterase type 5, is most commonly used to treat. The action of sildenafil is to relax smooth muscles and dilate blood vessels by increasing levels of cyclic guanosine monophosphate, which is achieved by inhibiting phosphodiesterase type 5. The mechanism that underpins its therapeutic benefits is the enhancement of blood flow to certain tissues (Kukreja et al. 2005). Previous experimental studies have demonstrated that sildenafil possesses significant antioxidant effects in diabetes-induced erectile dysfunction (Morano et al. 2007). Sildenafil has also been investigated in ischemia–reperfusion injury in ovarian tissue, and it was effective in preventing reperfusion injury after ischemia (Ganla et al. 2019; Incebiyik et al. 2015). Animal models demonstrated that sildenafil medication maintained primary follicle count and had no significant change in the secondary follicle count, ovarian size, or AMH level (Ergin et al. 2022). Sildenafil sustained normal concentrations of E2 and AMH, while inhibiting the elevation of FSH and LH, often observed in cisplatin-induced POF (Taskin et al. 2015). Side effects include headache, flushing, nasal congestion, visual disturbances, and, rarely, cardiovascular events, particularly in those with pre-existing heart disease (Ausó et al. 2021).

### Atorvastatin

Statins are a type of medication that are classed as 3-hydroxy-3-methyl-glutaryl coenzyme A reductase inhibitors (Stancu and Sima 2001). ATV has an anti-inflammatory (Fassett and Coombes 2013) and antioxidant effect (Crevar-Sakač et al. 2016). Low doses of ATV have been prescribed for treating hyperlipidemia. On the other hand, high doses can cause many complications, including nephrotoxicity (Nasri et al. 2016) and testicular injury (Klinefelter et al. 2014). On the other hand, ATV does not have any adverse effects on fertility or reproduction when it is administered at low doses (Dostal et al. 1996). An increase in the levels of estrogen and progesterone, a decrease in the levels of MDA, and an increase in cell viability are all characteristics of ATV that have been found to have a protective impact against CP-induced ovarian damage. Additionally, ATV was able to stabilize the ovarian histological structure while simultaneously lowering the positivity level of caspase-3 (Hamzeh et al. 2018). Main risks comprise myopathy, rhabdomyolysis (rare), elevated liver transaminases, and new-onset diabetes. ATV is contraindicated in pregnancy due to teratogenic potential (Thompson et al. 2016).

### Azilsartan

As an angiotensin II receptor antagonist, azilsartan has been shown to possess significant anti-inflammatory and antioxidant pharmacological effects (Perry 2012). Azilsartan treatment in an animal model of CP-induced POF demonstrated a potential protective effect via its anti-inflammatory effects, which was evidenced by increasing IL-10 levels and decreasing TNF-α levels. The antioxidant effect of azilsartan preserved the structure and number of ovarian follicles after CP exposure (Mahmood 2024). The most typical side effects are hypotension, dizziness, hyperkalemia, and rare angioedema (Lam 2011).

### Berberine

An isoquinoline alkaloid that is derived from natural sources is berberine or BBR. Strong antibacterial, anti-inflammatory, anti-hypoglycemic, and antioxidant effects are displayed by it (Tillhon et al. 2012; Li et al. 2014). It is mostly used to treat bacterial infections of the gastrointestinal system (Song et al. 2020). BBR has also been used to treat ulcers, diabetes, cancer, and cardiovascular diseases, as demonstrated in a number of earlier research (Ai et al. 2021; Liu et al. 2018b). Ovarian shrinkage, weight loss, and a reduction in ovarian follicular reserve are all features of CP, which also leads to hormonal imbalance. However, all these discrepancies were significantly improved by BBR therapy. Furthermore, BBR was shown to activate the Nrf2 pathway and inhibit the NF-kB pathway, hence reducing the buildup of ROS and mitochondrial dysregulation in ovarian tissues (Peng et al. 2023). BBR can lower FSH levels while concurrently promoting the production of AMH and estrogen to enhance ovarian function. This aids in preserving a steady hormonal balance (Peng et al. 2023). BBR is mostly associated with mild gastrointestinal discomfort at high doses (Prajwala et al. 2020**).**

### Curcumin

One naturally occurring phytochemical that is present in turmeric is called curcumin (CRC) (Marchiani et al. 2014), which has been found to have notable benefits as an anti-inflammatory, anti-cancer, and analgesic (Haanpää and Treede 2012; Beltran et al. 2007). These activities’ mechanisms are especially linked to their anti-inflammatory effects, which include modifying macrophage function by lowering the production of lysosomal enzymes, proteases, and arachidonic acid metabolites (Shehzad et al. 2013). Chemotherapy has been found to significantly affect ovarian reserve, causing abnormalities in hormonal shifts, increased tissue oxidative stress, and increased histological damage. However, the levels of oxidative stress, ovarian reserve indicators, and histological abnormalities analyzed were significantly reduced when CRC and CP were administered together (Meirow et al. 2010; Lopes et al. 2014). It has been demonstrated that the mechanisms behind the antioxidative and anti-inflammatory effects of CRC include NF-κB suppression, which reduces the production of inflammatory cytokines and inducible NOS (Menon and Sudheer 2007). Moreover, quinone oxidoreductase 1 and glutathione S-transferase gene expression and the enzymatic activity of NAD(P)H are also upregulated by CRC (Jaja-Chimedza et al. 2017). The oxidative stress created as a result of CP administration results in ovarian failure by causing apoptosis and preventing the nuclear and cytoplasmic development of oocytes (Liang et al. 2017). Tissue oxidative stress markers, including MDA, GSH, and SOD, were improved after CRC administration (Melekoglu et al. 2018). Furthermore, there was a significant decrease in FSH and LH levels and a significant increase in AMH and E2 levels following the administration of CRC (Melekoglu et al. 2018). CRC is associated with mild gastrointestinal discomfort at high doses. Curcumin may also cause allergic dermatitis in rare cases (Burgos-Morón et al. 2010).

### Quercetin

Many different plants and natural foods, such as tea, kale, apples, and onions, contain quercetin, a naturally occurring flavonoid (Suarez-Almazor et al. 1996). It exhibits potent antioxidant and anti-inflammatory properties (Boots et al. 2008). By alleviating oxidative damage and activating mitochondrial biogenesis via the peroxisome proliferator-activated receptor gamma coactivator 1-alpha (PGC1-α) pathway, this drug can alleviate mitochondrial dysfunction (Chen et al. 2022). Furthermore, quercetin has been shown to support the maintenance of ovarian function in CP-induced POF by preventing the pyroptosis process (Chen et al. 2022). The injection of quercetin shields the ovarian reserve from the ovarian damage brought on by CP by increasing blood levels of AMH and E2 while concurrently lowering levels of FSH and LH (Jiao et al. 2021). This resulted in an increase in ATP levels as well as PGC1-α mRNA and protein expression. Additionally, it was found to have anti-pyroptotic potential by suppressing the levels of NLRP3, caspase-1, GSDMD, and IL-1β in the granulosa cells (Biasizzo and Kopitar-Jerala 2020). It is well-tolerated, with rare instances of headache, tingling, or renal impairment at extremely high doses (Najafi et al. 2022).

All previously discussed protective agents are summarized in Table 1, which serves as a comparative table to illustrate the molecular target, experimental model, and key outcomes associated with each protective agent.

**Table 1:** Comparative effects of protective agents on cyclophosphamide-induced ovarian toxicity in preclinical models

Drug/agent	Molecular targets	Experimental model	Key outcomes	Reference (s)
**Buspirone**	• NF-κB/NLRP3/caspase-1 pathway • Nrf2/HO-1 and p-AMPK signaling • α-Klotho protein expression • Bax/Bcl-2/caspase-3 apoptotic axis	Female Wistar albino rats were administered a single intraperitoneal dose of 200 mg/kg of CP on the first day, followed by a Daily dosage of 8 mg/kg for the subsequent 14 days	• Ovarian oxidative stress markers suggested an improved antioxidant status, demonstrated by lower MDA and elevated SOD and GSH levels • Lowered inflammatory cytokines (↓ TNF-α, IL-1β, IL-18) • Inhibited apoptosis (↓ caspase-3, ↑ Bcl-2) • Restored folliculogenesis and ovarian structure • Improved hormone levels (↑ AMH, E2, ↓ FSH) • Attenuated histopathological abnormalities	Khallaf et al. 2025)
**Levomilnacipran**	• TLR4/p38-MAPK/NF-κB p65 • Caspase-3 • Klotho protein expression	Female Wistar albino rats received a single intraperitoneal dosage of 200 mg/kg of CP on the first day, followed by 8 mg/kg for the subsequent 14 days	• ↓ Pro-inflammatory cytokines (TNF-α, IL-6) • ↓ Apoptotic index • ↑ AMH, Klotho, and estradiol levels • Preservation of follicular integrity	Rofaeil et al. 2025)
**Cilostazol**	• Nrf2/HO-1 pathway • Increased cyclic adenosine monophosphate	Female rats, administered CP at a dosage of 150 mg/kg via intraperitoneal injection as a single treatment	• Ovarian tissue showed decreased MDA levels and increased SOD and GSH activity, indicating an enhanced antioxidant profile • Enhanced Nrf2/HO-1 gene expression • Reduced ovarian apoptosis	Abdel-Aziz et al. 2020)
**Diosmin**	• Anti-oxidative and anti-inflammatory pathways • Expression of miRNA-145 and its target genes VEGF-B and RGC32	Swiss albino rats were given a single intraperitoneal dosage of 200 mg/kg of CP on the first day, followed by 8 mg/kg for the subsequent 14 days	• Increased AMH, E2 levels • Reduced oxidative stress • Preserved ovarian histology • Decreased follicle atresia	Abogresha et al. 2021)
**Donepezil**	• TLR4/NF-κB/NLRP3 pathway • Pro-inflammatory cytokines	Female Swiss albino mice were administered CP as a single intraperitoneal dose of (75 mg/kg)	• Dose-dependent increase in AMH • Lowered TLR4/NLRP3/IL-6/TNF-α expression • Improved healthy follicle count	Zidan et al. 2024)
**LCZ696**	• TLR4/NF-κB/NLRP3 pathway	Female Wistar albino rats received a single intraperitoneal dosage of 200 mg/kg of CP on the first day, followed by 8 mg/kg for the subsequent 14 days	• Markers indicative of oxidative stress were altered, with decreased MDA levels and increased GSH and SOD activities • Suppressed inflammation (↓ TNF-α, IL-18, IL-1β, NF-κB) • Inhibited NLRP3/caspase-1 activation • Preserved ovarian reserve, improved follicle count, and histology • Restored hormonal balance (↑ estradiol, AMH, ↓ FSH, LH)	Khallaf et al. 2023)
**Melatonin**	• Activating the Hippo signal pathway • Downregulating caspase-3, Bax-mediated apoptosis	Female Sprague Dawley rats were given a single intraperitoneal dosage of 50 mg/kg of CP on the first day, followed by 8 mg/kg for the subsequent 15 days	• Preserved follicle count • Up-regulated cysteine-rich angiogenic inducer 61 and connective tissue growth factor at the mRNA and protein levels • inhibited large tumor suppressor 1, Mps1-One binder, and yes-associated protein phosphorylation	Xu et al. 2022a)
	Feng et al. 2022)	Female mice were administered CP as a single intraperitoneal injection at a dosage of 75 mg/kg	• Ovarian reserve testing and hormonal assays • Mitochondrial apoptosis pathways	• Inhibited ovarian apoptosis and maintained AMH expression
**Moxibustion**	• NF-κB/TLR4 pathway • NLRP3 inflammasome/caspase-1	Female Sprague Dawley rats were given a single intraperitoneal dosage of 50 mg/kg of CP on the first day, followed by 8 mg/kg for the subsequent 15 days	• Suppressed inflammation (↓ IL-18, IL-1β) • Inhibited NLRP3/caspase-1 activation • Restored hormonal balance (↑ estradiol; ↓ FSH, LH) • Downregulated NF-κB/TLR4 expression	Yin et al. 2023)
**Resveratrol**	• SIRT1/Foxo3a pathway • Anti-apoptotic: inhibits caspase-3/Bax	Female Sprague Dawley rats were given a single intraperitoneal dosage of 50 mg/kg of CP on the first day, followed by 8 mg/kg for the subsequent 14 days	• The expressions of SIRT1, Foxo3a were up-regulated and p53, caspase-3, and Bax were down-regulated • Restored hormonal balance and follicular count	Nie et al. 2021)
**Irbesartan**	• Oxidative stress and apoptosis	Female rats were administered CP as a single-dose treatment at a dose of 100 mg/kg	• Lowered TNF-α, MPO, and increased IL-10 levels • Decreased caspase-3, P53 • Improved AMH, estradiol, ovarian histology abnormalities	Abdel-Raheem et al. 2015)
**Mirtazapine**	• Oxidative stress and inflammation	Female rats were administered CP as a single-dose treatment at a dose of 150 mg/kg	• Measurements revealed lower NO and MDA levels along with elevated GPx and SOD activities and diminished MPO activity • Improved histopathological aberrations and follicular count	Khedr 2015)
**Sildenafil**	• AMH assay and morphometric study	Female rats were administered CP as a single dose treatment at a dose of 200 mg/kg	• Preserving primary follicle counts only • Increased AMH levels	Ergin et al. 2022)
**Atorvastatin**	• Oxidative stress, inflammation, and apoptosis	Female rats were administered CP as a single-dose treatment at a dose of 150 mg/kg	• Increased estrogen and progesterone levels • Mitigated acute inflammation, degenerative cells in stroma and follicles, stromal edema, vacuolization, atresia of the follicles, and congestion • Reduced immunoreactivity level of caspase-3	Hamzeh et al. 2018)
**Azilsartan**	• Oxidative stress and inflammation	Female rats were administered CP as a single-dose treatment at a dose of 200 mg/kg	• Increased IL-10 levels and decreased TNF-α levels • Preserved the structure and number of ovarian follicles	Mahmood xxxx)
**Berberine**	• Nrf2/SOD/CAT • NF-κB, IL-6, and IL-1β	Female rats were administered CP as a single-dose treatment at a dose of 120 mg/kg	• Activated the Nrf2/SOD/CAT pathway and inhibited the NF-kB pathway • Downregulated IL-6 and IL-1β	Peng et al. 2023)
**Curcumin**	• Oxidative stress markers • Hormonal and histopathological assessment	Female Wistar albino rats received a single intraperitoneal dosage of 200 mg/kg of CP on the first day, followed by 8 mg/kg for the subsequent 14 days	• Reductions in MDA levels alongside increases in GSH and SOD activities, suggesting an improved antioxidant profile • Decreased FSH and LH levels and significantly increased AMH and estrogen levels	Melekoglu et al. 2018)
**Quercetin**	• Expression of PGC1-α • NLRP3, caspase-1, and gasdermin D levels	Female rats were administered CP as a single-dose treatment at a dose of 90 mg/kg	• Upregulated PGC1-α mRNA and protein expression • Suppressed the levels of NLRP3, caspase-1, GSDMD, and IL-1β in the granulosa cells	Biasizzo and Kopitar-Jerala 2020)

The summarized findings in Table 1 highlight the diverse pharmacological and natural agents evaluated for protection against CP-induced ovarian damage. Notably, these agents consistently target a core set of interconnected molecular mechanisms, primarily oxidative stress, inflammation, and apoptosis, which collectively contribute to follicular depletion and POF. Oxidative stress emerges as a central pathogenic feature, with CP metabolism generating ROS that disrupt redox balance (Khallaf et al. 2023). Protective agents commonly enhance the antioxidant defense system, predominantly via activation of the Nrf2/HO-1 pathway, leading to upregulation of phase II antioxidant enzymes such as SOD and GSH (Gao et al. 2023). For example, cilostazol, berberine, LCZ696, and buspirone demonstrated significant restoration of oxidative parameters, reducing lipid peroxidation and mitigating mitochondrial dysfunction. This antioxidative effect is critical for preserving GC viability and follicular integrity.

Inflammatory signaling is intricately linked with oxidative stress, where DAMPs released from injured ovarian cells activate TLR4 and downstream NF-κB and NLRP3 inflammasome pathways. This results in enhanced production of pro-inflammatory cytokines, including TNF-α, IL-6, and IL-1β (Rofaeil et al. 2025). Agents such as donepezil, levomilnacipran, and moxibustion suppress these pathways by inhibiting TLR4/NF-κB activation and NLRP3 inflammasome assembly, thereby alleviating inflammation and subsequent pyroptosis.

Apoptotic mechanisms are regulated through a delicate balance of pro- (Bax, caspase-3) and anti-apoptotic (Bcl-2) proteins, governing GC survival. Apoptotic cascades are upregulated after CP exposure, culminating in follicular loss (Khallaf et al. 2025). Several agents, including resveratrol, quercetin, and levomilnacipran, modulate apoptosis-related protein expression and caspase activity.

Particularly, the activation of SIRT1, an NAD⁺-dependent deacetylase with anti-apoptotic and anti-inflammatory properties, was noted to play a key role in mitigating GC apoptosis and promoting mitochondrial protection. Several agents also exhibit engagement with additional or complementary molecular pathways that contribute to ovarian protection (Xiu et al. 2023). For instance, levomilnacipran’s effects are associated with upregulation of the anti-aging protein α-Klotho, which has downstream regulatory effects on oxidative stress and inflammation. Melatonin’s protective role involves modulation of the Hippo signaling pathway and regulation of autophagy via the PI3K/AKT/mTOR axis. Buspirone additionally activates AMPK, supporting metabolic homeostasis. These auxiliary mechanisms highlight the pleiotropic actions of some protective agents and suggest potential synergistic therapeutic targets.

While numerous pharmacological and naturally derived agents demonstrated promising protective effects against CP-induced ovarian injury in animal models, a balanced interpretation of these findings necessitates consideration of various study limitations.

Many preclinical studies suffer from small sample sizes that limit statistical power. Detailed descriptions of randomization and blinding procedures are often missing, raising concerns about potential selection and observer biases. The heterogeneity in animal species, strain, and age adds variability that complicates cross-study comparisons (Spears et al. 2019). Experimental protocols vary widely, with differences in CP dosing schedules, timing, and duration of protective agent treatment, and choice of endpoints (Kim and You 2021). Most studies focus on biochemical markers (e.g., oxidative stress parameters, inflammatory cytokines) and histological assessments of ovarian tissue. Functional outcomes such as fertility restoration and long-term ovarian reserve preservation are infrequently reported. Short follow-up periods hinder assessment of sustained effects (Ogunro and Ofeniforo 2024). The reliance on surrogate endpoints limits the clinical relevance of findings. Additionally, animal models differ significantly from humans in CP metabolism and reproductive endocrinology (Ramirez et al. 2019). These differences challenge the direct translation of preclinical results. The wide range of CP dosages used in models, some exceeding clinically relevant exposures, further complicates relevance to patient care. Addressing these limitations requires rigorous study design with adequately powered cohorts, clearly documented randomization and blinding, and standardized CP and protective agent dosing paradigms. Incorporating multidimensional outcome measures, including molecular, histological, hormonal, and fertility metrics, and longer-term follow-up, will enhance understanding of protective efficacy and safety.

The protective effects observed in different animal models are highly dependent on the specific experimental protocols employed, particularly concerning the dose, timing, and administration route of the tested agents. Variations in the administered dose can profoundly influence not only the magnitude of observed effects but also the potential for toxicity or subtherapeutic responses, thereby complicating cross-study comparisons. Similarly, the timing of intervention, whether administered before, concurrent with cyclophosphamide, may determine the extent of protection conferred, as it can affect the agent’s ability to precondition tissue, interrupt ongoing damage, or promote recovery. Moreover, the route of administration (e.g., intraperitoneal, intravenous, oral, or local injection) can substantially alter agent bioavailability, distribution, and pharmacodynamics, which in turn impacts tissue targeting and effectiveness. These variables, which often differ widely across studies, likely contribute to the heterogeneity observed in outcomes and may restrict the generalizability of results. Future research should prioritize systematic evaluation of these parameters to optimize protective protocols and enhance the translational relevance of experimental findings.

Additionally, a limitation of the present study is its reliance on available evidence from the preclinical level, which reported associative biochemical markers (e.g., SOD, GSH) to infer mechanistic protection. Although these markers are well-established indicators of oxidative stress and cellular status, their improvement may be correlative rather than causative regarding functional protection (Ghezzi et al. 2017). Future investigations utilizing targeted mechanistic interventions and causal experimental approaches (e.g., specific pathway inhibitors or genetic models) are warranted to clarify direct cause-and-effect relationships.

## Emerging non-pharmacological therapies: stem cell therapy and exosome-based therapy in POF

### Stem cell therapy

Contemporary interventions such as HRT mitigate menopausal symptoms but carry considerable risks, including breast and endometrial cancer with prolonged usage, thromboembolism, and inability to reinstate ovarian endocrine function or fertility (Xinyue et al. 2025). Ovulation induction demonstrates limited efficacy in autoimmune-related cases, underscoring the pressing necessity for regenerative strategies aimed at restoring ovarian function. Stem cells, noted for their self-renewal and differentiation capabilities, may potentially reverse ovarian dysfunction by facilitating folliculogenesis, diminishing GC apoptosis, and augmenting ovarian angiogenesis. Their action may occur through direct differentiation or predominantly via paracrine mechanisms that release growth factors and cytokines beneficial to ovarian tissue regeneration (Kim and Kim 2024; Ali et al. 2022). Prevalent sources include bone marrow, adipose tissue, adipose-derived stem cells, umbilical cord, menstrual blood, and synovial membrane. Autologous transplantation, utilizing the patient’s tissue, is preferred to mitigate immunological responses (Ali et al. 2022; Wang et al. 2022). Preclinical animal studies demonstrated enhancements in hormone levels (e.g., estradiol), follicle counts, and ovarian mass in ovarian injury caused by CP. Numerous minor clinical investigations have indicated elevated AMH, reduced FSH, improved antral follicle count (AFC), and recovery of menstruation, as well as instances of pregnancy after stem cell therapy (Kim and Kim 2024; Wang et al. 2022; Umer et al. 2024a). Meta-analyses have validated considerable enhancement in ovarian function measures and reproductive outcomes following stem cell transplantation (Umer et al. 2024a, 2024b). Mesenchymal stem cells (MSCs) release cytokines, growth factors, and anti-apoptotic proteins that diminish GC apoptosis, promote angiogenesis, and regulate inflammation. This alters the ovarian microenvironment (Zhou et al. 2023). MSCs can develop into germ cell-like cells, theca cells, and oocyte-supporting stromal cells in response to ovarian niche signals, therefore replenishing depleted cell populations (Xinyue et al. 2025). MSCs inhibit pro-inflammatory cytokines (TNF-α, IL-6, IL-17) and enhance T-regulatory cell activity, mitigating autoimmune factors associated with POF (Wu et al. 2024). Adipose-derived MSCs are harvested through a minimally invasive procedure. Studies in rodent models demonstrated their ability to enhance follicular counts and restore estrous cycles by activating the SIRT3/SOD2 pathway, thereby decreasing oxidative stress (Xinyue et al. 2025; Zhou et al. 2023). MSCs from the umbilical cord enhanced proliferative ability; clinical investigations demonstrated increased ovarian reserve, as indicated by elevated AMH levels and improved pregnancy rates. Secretome abundant in miR-21-5p enhances angiogenesis (Xinyue et al. 2025; Tan et al. 2024). Despite promising findings, stem cell therapy encounters obstacles such as limited sample sizes in trials, the absence of standardized protocols, ethical dilemmas, and the necessity for long-term safety data (Kim and Kim 2024; Wang et al. 2022).

#### Bone marrow mesenchymal stem cells (BMSCs)

BMSCs improved POF via a series of coordinated mechanisms, beginning with targeted migration and homing to damaged ovarian tissue. The localization of BMSCs within ovarian stroma (hilum, medulla, cortex) is mediated by chemokine receptors such as C-X-C chemokine ligand-8 and hepatocyte growth factor (HGF). BMSCs do not differentiate into oocytes; rather, they exert therapeutic effects primarily through paracrine signaling. These cells secrete various bioactive factors, including VEGF, insulin-like growth factor-1 (IGF-1), HGF, basic fibroblast growth factor (bFGF), and transforming growth factor (TGF-β), which collectively facilitate ovarian recovery through multiple pathways (Huang et al. 2022). Secreted IGF-1 and VEGF inhibit GC apoptosis through the upregulation of Bcl-2 and proto-oncogene (c-myc), alongside the suppression of Bax and cyclin-dependent kinase inhibitor 1 A (p21) (Hu et al. 2024). MicroRNA-21 (miR-21) amplifies this effect through the targeting of phosphatase and tensin homolog deleted on chromosome ten, thus Maintaining follicular integrity. HGF and bFGF decrease collagen deposition and fibroblast proliferation, thereby alleviating ovarian fibrosis, which is a characteristic of POF pathogenesis. VEGF and HGF work together to enhance neovascularization, thereby restoring blood flow to ischemic ovarian tissue. Angiogenin and membrane type 1-matrix metalloproteinase enhance nutrient delivery and tissue repair. BMSCs inhibit pro-inflammatory cytokines, such as TNF-α and IL-1, through the secretion of prostaglandin E2, indoleamine-2,3-dioxygenase, and TGF-β. They simultaneously enhance regulatory T-cell (Treg) activity and facilitate macrophage reprogramming to an anti-inflammatory phenotype (IL-10), thereby reestablishing Th17/Treg ratios that are altered in autoimmune POF (He et al. 2018).

#### Human umbilical cord mesenchymal stem cells

Human umbilical cord mesenchymal stem cells (HUCMSCs) primarily alleviate POF through paracrine mechanisms rather than direct differentiation. These cells release various bioactive factors, such as cytokines, growth factors, and exosomes, which together facilitate tissue repair and regulate the immune system. The secreted “conditioned medium” reduces GCs’ apoptosis by activating survival pathways, including PI3K/AKT, and regulating factors such as granulocyte colony–stimulating factor (Deng et al. 2021). Additionally, it enhances GCs’ proliferation, which is essential for follicle Maturation. HuCMSCs enhance ovarian function by restoring T Helper 1/T Helper 2 cytokine balance and modulating uterine natural killer cell expression, thereby improving endometrial receptivity. The overall outcome includes an increase in healthy follicles, restoration of normal hormone levels, and normalization of the estrous cycle (Shareghi-Oskoue et al. 2021). HUCMSCs have a low risk of immune rejection, are easily collected, and pose fewer ethical and tumorigenicity concerns than other stem cell sources.

#### Adipose-derived mesenchymal stromal cells

Adipose-derived mesenchymal stromal cells (ADMSCs) improve POF through the restoration of ovarian function by establishing a regenerative microenvironment, rather than through direct differentiation into oocytes. Transplanted autologous adipose-derived mesenchymal stromal cells are thought to exert therapeutic effects through paracrine signaling by secreting bioactive molecules, including VEGF, placental growth factor (PGF), and TGF-β (Takehara et al. 2013). These molecules promote angiogenesis, decrease GCs’ apoptosis, and modulate the local immune environment. These actions facilitate the repair of compromised ovarian niches, enhance the survival of existing follicles, and may activate dormant ovarian stem cells found in the tunica albuginea. This activation can result in the differentiation of OSCs into germ cells and primitive GC nests, ultimately contributing to the formation of new primordial follicles. This process facilitates follicular development and ovarian function, leading to enhanced hormonal profiles, including reduced FSH levels, and, in certain instances, the restoration of menstruation, as evidenced by clinical studies (Mashayekhi et al. 2021). Furthermore, ADMSCs alleviate oxidative damage in ovarian tissues via upregulation of protective pathways, including SIRT1 and FOXO1 (Hu et al. 2024).

#### Human amniotic epithelial cells

Human amniotic epithelial cells (hAECs) mitigate POF via a complex mechanism that includes migration, differentiation, and the restoration of the ovarian microenvironment, as evidenced in mouse models of chemotherapy-induced POF (Zhang et al. 2020). After intravenous transplantation, hAECs migrate to damaged ovarian tissue, where they first integrate into the ovarian stroma and later localize near follicles, ultimately encircling oocytes. hAECs specifically differentiate into GCs, as confirmed by the presence of human-specific nuclear antigen and human follicle-stimulating hormone receptor expression in recipient ovaries (Zhao et al. 2024). There is no differentiation into germ cells, indicated by the lack of human Markers in oocytes. The transplanted cells facilitate essential somatic-germ cell interactions by offering structural and Functional support to developing follicles. Notably, AMH expression reemerges in primary follicles as early as 14 days post-transplantation, with a progressive increase over time, signifying the restoration of ovarian function. The mechanism entails the trans-differentiation of human amniotic epithelial cells into functional GCs, which facilitate oocyte growth and follicular development. Concurrently, this process reactivates host germ line stem cells within the damaged ovary, effectively restoring the complete folliculogenesis process from primordial to antral follicle stages, without any indication of germ cell differentiation from the transplanted hAECs (Wang et al. 2013b).

#### Amniotic fluid stem cells

Amniotic fluid stem cells (AFSCs) alleviate POF mainly by inhibiting follicular atresia and promoting healthy follicle development, rather than by directly differentiating into ovarian cell lineages. This is evidenced by studies using chemotherapy-induced POF mouse models in which AFSCs were transplanted into damaged ovaries (Lai et al. 2013). Post-transplantation, AFSCs integrate into ovarian tissue and exert therapeutic effects via paracrine signaling mechanisms. They secrete growth factors (e.g., TGF-β, VEGF, glia cell-derived neurotrophic factor) and exosomes containing beneficial miRNAs (such as miR-10a and miR-369-3p) that support the survival and proliferation of GCs and inhibit apoptosis (Xiao et al. 2016). Research indicates that they secrete proangiogenic soluble factors that facilitate the recruitment of endothelial progenitors and improve vascularization in the damaged ovarian microenvironment. Instead of differentiating into primitive oocytes as previously hypothesized (given the absence of progeny from transplanted AFSCs), these cells operate by modulating key cellular processes related to follicular atresia, specifically by regulating the expression of genes associated with cell death and survival in GCs, which are crucial for follicular development. AFSCs exhibit expression of OCT4 and additional pluripotency markers, while preserving a rapid self-renewal capacity, maintaining a normal karyotype in long-term culture, and demonstrating low immunogenicity, which enables them to evade immune rejection. These characteristics render them especially appropriate for therapeutic applications. Their mechanism entails the interruption of apoptotic pathways in GCs, which have been previously identified as a critical factor in follicular atresia. This approach fosters a regenerative microenvironment that sustains existing follicles, ultimately restoring ovarian function, preserving fertility, and averting further degeneration of the ovarian reserve in conditions of POF (Xiao et al. 2014).

#### Ovarian epithelial-like cells

Ovarian epithelial-like cells improve POF via a complex mechanism that includes tissue integration, reduction of fibrosis, and restoration of hormonal balance (Bukovsky and Caudle 2012). This was evidenced in mouse models of chemotherapy-induced POF, where estrogen-sensitive ovarian epithelial (OSE)-like cells derived from human-induced pluripotent stem cells were transplanted into damaged ovaries. Post-transplantation, microRNA-17-3p-induced OSE-like cells demonstrate successful survival and proliferation within the POF ovarian microenvironment for a minimum of 14 days. These cells integrate into the ovarian tissue, modulating the expression of essential cellular markers by significantly increasing epithelial markers (cytokeratin 7) and estrogen receptors (ERβ), while concurrently decreasing mesenchymal markers (fibronectin and vimentin), thus reversing the ovarian fibrosis associated with POF. The molecular reprogramming results in structural enhancements such as increased ovarian weight, decreased atretic follicle counts, and heightened mature follicle numbers, with transplanted cells successfully reinstating the ovarian microenvironment essential for follicular development. The therapeutic mechanism includes the reactivation of hormonal signaling pathways, demonstrated by significantly elevated plasma E2 levels in treated mice over time. Additionally, the inherent capacity of OSE-like cells to express various growth factors, such as epidermal growth factors, plays a role in tissue repair and regeneration. These cells operate via paracrine signaling instead of differentiating into new oocytes, thereby establishing a supportive microenvironment that prevents existing follicles from atresia and facilitates their development during folliculogenesis. This process alleviates the pathological manifestations of POF without modifying FSH levels, while effectively reinstating the estrogenic environment necessary for normal ovarian function (Liu et al. 2013).

### Exosome-based therapy

Exosomes are nanoscale extracellular vesicles released by stem cells and other cell types, containing proteins, RNAs, and microRNAs that influence biological functions. Exosome therapy utilizes these vesicles to facilitate tissue repair and anti-apoptotic effects without the incorporation of complete cells, perhaps providing a safer, cell-free alternative to stem cell transplantation (Kim and Kim 2024; Ali et al. 2022). Investigations into exosome therapy for POF are predominantly in the preclinical phase, with encouraging outcomes in enhancing ovarian function through the mitigation of oxidative stress and apoptosis, as well as the stimulation of angiogenesis and follicle maturation (Kim and Kim 2024; Wang et al. 2022). Exosomes mitigate POF by exerting anti-apoptotic activities, such as exosomal miR-21-5p inhibiting PTEN and caspase-3, hence decreasing GC apoptosis. Hypoxic ADSC-exosomes enhance miR-21-3p/miR-126-5p expression, hence activating the PI3K/AKT pathway to facilitate GC survival. Promotion of angiogenesis by miR-126-3p from ADSC-exosomes targets PIK3R2, hence improving vascularization in rat ovaries. miR-125a-3p enhances HUVEC migration through PTEN suppression. Furthermore, the mitigation of oxidative stress through miR-378 in ADSC-exosomes diminishes ROS levels in human adult low calcium temperature (HaCaT) cells, whereas the SIRT3/SOD2 pathways enhance mitochondrial activity under high-glucose conditions (Xinyue et al. 2025; Zhou et al. 2023).

#### Human amniotic epithelial cell-derived exosomes

Human amniotic epithelial cell-derived exosomes present a viable therapeutic strategy for alleviating POF induced by chemotherapy, leveraging various synergistic mechanisms focused on their bioactive microRNA content. The exosomes, exhibiting a characteristic cup-shaped morphology of approximately 100 nm in diameter and positive for exosomal markers Alix, CD63, and CD9, are secreted by human amniotic epithelial cells, which demonstrate stem cell–like properties such as low immunogenicity and immunomodulatory capabilities (Zhang et al. 2019). In mouse models of chemotherapy-induced POF, the transplantation of human amniotic epithelial cell-derived exosomes administered both directly into damaged ovaries and systemically substantially enhances ovarian function by augmenting the quantity of healthy primordial, primary, secondary, and mature follicles, which are otherwise diminished by chemotherapeutic agents. The main mechanism by which hAEC exosomes facilitate their restorative effect is through the inhibition of GCs’ apoptosis, a characteristic of chemotherapy-induced ovarian damage (Geng et al. 2023). Exosomes facilitate the transfer of functional miRNAs, particularly miR-1246, into GCs. Within these cells, the miRNAs downregulate apoptotic Markers, including cleaved caspase 3 and pro-apoptotic proteins such as Bax, while simultaneously upregulating anti-apoptotic proteins like Bad and Bcl2. This molecular modulation maintains GCs’ viability and supports follicular integrity. Simultaneously, hAEC exosomes increase the expression of cumulus expansion-related genes, including hyaluronic acid synthase 2 and pentraxin 3, which are essential for oocyte nourishment and follicular development. In addition to promoting cellular apoptosis, hAEC exosomes mitigate acute vascular injury in the ovary caused by chemotherapy. This is evidenced by the enhanced proliferation and maintenance of endothelial cells (CD34-positive), which are essential for the sustenance of ovarian blood supply and follicle health. Moreover, these exosomes partially inhibit premature activation and depletion of primordial follicles by modulating the PI3K/AKT/mTOR signaling pathway, which is typically hyperactivated by chemotherapy, resulting in follicle “burnout.” Following treatment with hAEC exosomes, there is a reduction in the phosphorylation levels of critical proteins, including Akt and FoxO3a, which contributes to the preservation of ovarian reserve. The miRNA cargo in hAEC exosomes targets various biological pathways, including phosphatidylinositol signaling, PPARγ signaling, AMPK pathways, and apoptotic processes, as demonstrated by microarray and bioinformatics analyses. The pathways collectively contribute to metabolic regulation, anti-inflammatory effects, and tissue remodeling, thereby enhancing ovarian restoration. Transcriptomic analyses of treated ovarian tissue confirm the reversal of gene expression alterations induced by chemotherapy, aligning with enhanced metabolic functions and diminished inflammatory responses. In vitro experiments demonstrate the effective internalization of hAEC exosomes by human granulosa tumor cells and their ability to transfer miRNAs that inhibit chemotherapy-induced apoptosis and restore cellular function. The continuous release of miRNAs, including miR-1246 and miR-21-5p, correlates with the suppression of apoptosis-related proteins, highlighting the therapeutic potential of exosomal miRNA cargo (Geng et al. 2023).

#### Bone marrow mesenchymal stem cell-derived exosomes

Bone marrow mesenchymal stem cell–derived exosomes (BMSC exosomes) alleviate POF primarily by delivering specific miRNAs, particularly miR-144-5p, which demonstrates anti-apoptotic effects on ovarian GCs affected by chemotherapy (Yang et al. 2020). In POF models induced by CP, treatment with BMSC exosomes significantly restores ovarian function, as indicated by the normalization of estrous cycles, an increase in healthy basal and sinus follicles, and improved serum hormone profiles, including elevated E2 and AMH, alongside decreased levels of FSH and LH. BMSC exosomes are internalized by damaged GCs, wherein the transferred miR-144-5p directly targets the tumor suppressor gene PTEN, which negatively regulates the PI3K/AKT signaling pathway (Huang et al. 2022). The inhibition of PTEN by miR-144-5p results in the activation of the PI3K/AKT signaling pathway, thereby enhancing cell survival and reducing apoptosis. Decreased expression of apoptotic Markers, including caspase 3 and caspase 9, along with reduced rates of GC apoptosis in vitro and in vivo, following exosome treatment, supports this observation. Functional assays confirm that the overexpression of miR-144-5p enhances the protective effects of BMSC exosomes against GC apoptosis, whereas the inhibition of miR-144-5p reverses these benefits. Blocking exosome release from BMSCs significantly reduces their ability to prevent GC apoptosis, indicating that exosomal miR-144-5p plays a crucial role in the therapeutic effect. BMSC-derived exosomal miR-144-5p preserves GCs’ viability, maintains follicular integrity, and prevents follicular atresia, thereby facilitating ovarian regeneration and restoring fertility. This exosome-based mechanism offers a cell-free regenerative strategy that addresses challenges linked to direct stem cell transplantation, including immune rejection and tumorigenicity, and underscores the potential use of BMSC exosomes as an innovative therapeutic approach for chemotherapy-induced POF (Xinyue et al. 2025).

#### Exosomes derived from human adipose mesenchymal stem cells

Exosomes originating from human adipose mesenchymal stem cells (hADSC-Exos) alleviate POF via a complex molecular mechanism that primarily involves the modulation of the homologs of Sma and Mad proteins (SMAD) signaling pathway (Huang et al. 2018). Small extracellular vesicles encompass bioactive molecules that, when administered in a POF mouse model or cocultured with GCs from POF patients, facilitate ovarian recovery by increasing follicle numbers at all developmental stages (primordial, primary, secondary, and antral) and normalizing hormone levels, including E2, AMH, and FSH. hADSC-Exos promote the proliferation of GCs and significantly reduce their apoptosis at the cellular level. This is accomplished through the upregulation of key SMAD proteins SMAD2, SMAD3, and SMAD5, serving as intracellular effectors of the TGF-β signaling pathway, which is crucial for follicular development, oogenesis, and GCs’ function. The elevation of SMAD signaling, prompted by hADSC-Exos at both mRNA and protein levels, results in the downregulation of essential apoptosis-related genes, such as Fas, Fas ligand, caspase-3, and caspase-8. The repression of apoptosis genes enhances the survival and functionality of ovarian cells, thereby aiding in the restoration of ovarian morphology and function. Additionally, the knockdown of SMAD proteins via RNA interference leads to an upregulation of apoptosis markers, thereby confirming that the therapeutic effects of hADSC-Exos are mediated through the SMAD pathway. hADSC-Exos function as modulators of SMAD signaling, inhibiting GC apoptosis, enhancing proliferation, and restoring endocrine function, thus effectively alleviating POF. This mechanism establishes hADSC-Exos as a viable cell-free regenerative therapy for POF, offering benefits such as low immunogenicity and enhanced safety compared to direct stem cell transplantation (Huang et al. 2018).

## Conclusion and future perspectives

A significant challenge to women’s health, POF can cause infertility, malfunction of the endocrine system, and long-term systemic issues. It is particularly concerning that CP might cause harm to the ovaries since ovarian tissue is susceptible to chemotherapy medicines. As our knowledge of its causes has grown, including oxidative stress, inflammation, and apoptosis, protective treatments have become feasible. So, to develop new therapies to lessen the effects of CP-induced ovarian damage, further studies are needed to assess other signaling molecular pathways that may be involved. A major challenge in translating preclinical data to clinical applications lies in the significant differences between animal models and human physiology, including variations in ovarian physiology, follicle dynamics, hormonal regulation, and drug metabolism can lead to divergent responses between rodents and humans. For instance, the rate of primordial follicle depletion, the sensitivity to chemotherapeutic injury, and the capacity for ovarian repair differ substantially between species. Moreover, CP dosing regimens utilized in experimental studies often differ markedly from those used in clinical practice. Animal models frequently employ single high doses or repeated supraphysiological doses (e.g., 100–200 mg/kg, i.p.) to induce rapid ovarian failure, while human chemotherapy protocols typically involve lower fractionated doses administered over longer periods. These discrepancies may exaggerate the extent and rapidity of ovarian damage in preclinical settings, and do not always accurately predict clinical outcomes such as fertility preservation or recovery potential observed in women receiving CP. Future research should address these gaps by adopting animal models and dosing regimens that closely mimic human clinical exposure, possibly using chronic, fractionated CP protocols; reporting and discussing the clinical relevance of animal doses, schedules, and endpoints for each protective agent; and prioritizing multi-dimensional outcome measures, including fertile potential and live birth rates over surrogate biochemical or histological markers. The integration of more clinically relevant models and study designs will strengthen the translational impact of basic research and guide the development of effective ovarian protective strategies for women undergoing CP chemotherapy.

## Data Availability

All source data for this work (or generated in this study) are available upon reasonable request.

## Abbreviations

AFC: Antral follicle count
AFSCs: Amniotic fluid stem cells
AMH: Anti-Mullerian hormone
AMPK: AMP-activated protein kinase
ANCA: Anti-neutrophil cytoplasmic antibody
Apaf-1: Apoptotic protease activating factor-1
AP-1: Activator protein 1
ASC: Apoptosis-associated speck-like protein
ATV: Atorvastatin
ADSCs: Adipose-derived stem cells
ADMSCs: Adipose-derived mesenchymal stromal cells
ATP: Adenosine triphosphate
Bax: Bcl-2 associated X protein
BBR: Berberine
Bcl-2: B-cell lymphoma 2
BID: BH3 interacting domain death agonist
BMSCs: Bone marrow mesenchymal stem cells
BUS: Buspirone
bFGF: Basic fibroblast growth factor
CAT: Catalase
CNS: Central nervous system
COX-2: Cyclooxygenase-2
CP: Cyclophosphamide
CR: Curcumin
DAMPs: Damage-associated molecular patterns
DISC: Death-inducing signaling complex
E2: Estradiol
FDA: Food and Drug Administration
Foxo3: Forkhead box O3
FSH: Follicle-stimulating hormone
GnRH: Gonadotropin-releasing hormone
GC: Granulosa cell
GPx: Glutathione peroxidase
GSDMD: Gasdermin D
GSH: Glutathione
hAECs: Human amniotic epithelial cells
HaCat: Human adult low calcium temperature cells
HO-1: Heme oxygenase-1
HRT: Hormone replacement therapy
HUVEC: Human umbilical vein endothelial cell
HUCMSCs: Human umbilical cord mesenchymal stem cells
HGF: Hepatocyte growth factor
IL-1β: Interleukin-1 beta
IL-6: Interleukin-6
IL-18: Interleukin-18
IGF-1: Insulin-like growth factor-1
IRAK: IL-1 receptor-associated kinase
IRB: Irbesartan
KEAP1: Kelch-like ECH-associated protein 1
LCZ696: Sacubitril/valsartan (drug combination)
LVM: Levomilnacipran
MAPK: Mitogen-activated protein kinase
MDA: Malondialdehyde
miRNA: MicroRNA
MPO: Myeloperoxidase
MSCs: Mesenchymal stem cells
MTZ: MSCs
mTOR: Mammalian target of rapamycin
MyD88: Myeloid differentiation primary response 88
NAD⁺: Nicotinamide adenine dinucleotide (oxidized form)
NF-κB: Nuclear factor kappa-light-chain-enhancer of activated B cells
NLRP3: NOD-like receptor family pyrin domain-containing 3 inflammasome
NO: Nitric oxide
Nrf2: Nuclear factor erythroid 2-related factor 2
ONOO⁻: Peroxynitrite
PGC1-α: Peroxisome proliferator-activated receptor gamma coactivator 1-alpha
PI3K: Phosphatidylinositol 3-kinase
PKB: Protein kinase B (also known as Akt)
POF: Premature ovarian failure
PPAR-γ: Peroxisome proliferator-activated receptor gamma
PTEN: Phosphatase and tensin homolog
RES: Resveratrol
RGC32: Regulator of cell cycle
ROS: Reactive oxygen species
SIRT1: Silent information regulator-1 (sirtuin 1)
SNRI: Serotonin and norepinephrine reuptake inhibitor
SOD: Superoxide dismutase
TIR: Toll-interleukin 1 receptor
TIRAP: Toll-interleukin 1 receptor (TIR) domain-containing adaptor protein
TLR4: Toll-like receptor 4
TNF-α: Tumor necrosis factor-alpha
TRAF6: TNF receptor-associated factor 6
TRAM: Translocating chain-associated membrane protein
TRIF: TIR-domain-containing adapter-inducing interferon-β
TGF-β: Transforming growth factor
VEGF-B: Vascular endothelial growth factor B
Wnt: Wingless-related integration site (signaling pathway)
